# First genome report of *Oudemansiella apalosarca* and comparative transcriptomics on fruiting body formation under different light conditions

**DOI:** 10.3389/ffunb.2026.1763719

**Published:** 2026-02-10

**Authors:** Qi Gao, Sai Wei, Yangyang Fan, Dong Yan, Yu Liu, Shuang Song, Hongxin Hu, Gawesha Yasapala, K L Wasantha Kumara, Shouxian Wang

**Affiliations:** 1 Institute of Plant Protection, Beijing Academy of Agriculture and Forestry Sciences, Beijing Engineering Research Center for Edible Mushroom, Beijing, China; 2 Department of Agricultural Biology, Faculty of Agriculture, University of Ruhuna, Kamburupitiya, Sri Lanka

**Keywords:** cap coloration, genome, light, *Oudemansiella apalosarca*, transcriptome, tyrosinase

## Abstract

*Oudemansiella apalosarca*, a newly identified edible fungus, exhibits industrial cultivation potential because of its short production cycle and lack of a casing layer. However, the absence of genomic data has hampered its development and varietal enhancement. This study reports the first genome sequence of *O. apalosarca*, comprising 53.13 Mb across 27 scaffolds and 14,650 protein-coding genes, with superior scaffold N50 and BUSCO values compared to those of other *Oudemansiella* genomes. Phylogenetic analysis of single-copy orthologous proteins from 25 fungal genomes revealed close relations to *O. raphanipes* and *Mucidula mucidula*, with significant protein collinearity within the Physalacriaceae family. Cultivation in complete darkness yielded pure white, small-cap, and long-stipe fruiting bodies, indicating industrial advantages. Differential transcriptome analysis of the cap and stipe under varying light conditions identified key genes and pathways regulating phenotypic changes. Kyoto Encyclopedia of Genes and Genomes (KEGG) assessment and gene set enrichment analysis (GSEA) revealed significant negative regulation of DNA replication pathway genes in the cap, with the downregulation of 10 cell cycle and mismatch repair genes. Genes related to cell wall formation and carbon metabolism were upregulated, thus promoting stipe elongation. The tyrosine metabolism pathway influenced cap coloration, with tyrosinase identified as a multicopy gene. Phylogenetic analysis revealed diverse evolutionary origins. Key tyrosinase-related genes A6_A10477 and A6_A09603 were overexpressed in the light, revealing their role in melanin formation. In summary, this study provides genomic resources for *O. apalosarca* breeding improvement and elucidates light-induced regulatory mechanisms in its development, thereby providing theoretical and technical support for industrial applications.

## 1 Introduction

With advancements in mushroom breeding and cultivation technology and evolving consumer demand, an increasing number of mushroom varieties are moving toward industrialized and commercialized cultivation. In China, the primary industrially cultivated species include *Flammulina velutipes*, *Pleurotus eryngii*, and *Hypsizygus marmoreus* (Changtian et al., 2019; Li and Xu, 2022). However, the industrial mushroom sector faces challenges owing to the proliferation of enterprises producing the same species, resulting in high production density and an oversupply structure that fails to meet consumer demand for diverse mushroom products (Changtian et al., 2019). Diversifying mushroom breeding is crucial (Dong et al., 2024). Therefore, the development of new mushroom varieties that are suitable for industrial production is essential.

*Oudemansiella apalosarca*, a member of the Physalacriaceae family within the Agaricomycetes, Basidiomycota (Dulay, 2023), is part of the rich *Oudemansiella* resources of China, which include species such as *O. radicata*, *O. raphanipes*, *O. brunneomarginata*, and *O. submucida* (Hao et al., 2016). These species predominantly occur in Yunnan, Hainan, Heilongjiang, and Jilin provinces (Hao et al., 2016). *Oudemansiella* species are valued as edible and medicinal mushrooms, characterized by tender flesh, crispy texture, and rich flavor compounds (Xia et al., 2022). They are nutritionally dense, contain proteins, amino acids, polysaccharides, trace elements, and dietary fiber, and exhibit medicinal properties, including antihypertensive and anticancer effects (Dulay, 2023). Over the years, we have focused on the collection and domestication of *Oudemansiella* germplasm resources, thereby establishing a germplasm bank comprising over 500 strains across 10 species. We have successfully domesticated and bred several varieties, including *O. apalosarca* and *O. brunneomarginata*. Notably, the *O. apalosarca* strain BIPP21200004, a new edible fungus variety with independent intellectual property rights in China, exhibiting a biological efficiency of up to 122% (Cui et al., 2021). Compared to other *Oudemansiella* species, such as the widely cultivated *O. raphanipes*, *O. apalosarca* has a brief production cycle, higher yield potential, and does not require soil covering (Cui et al., 2021). Soil covering in *O. raphanipes* cultivation often results in contamination with miscellaneous fungi and necessitates the labor-intensive cleaning of fruiting bodies, thereby reducing efficiency (Qin et al., 2022). These attributes render *O. apalosarca* well suited for industrial mushroom cultivation, addressing the demands for high yield, short production cycles, and labor savings. To enhance the production efficiency of large-scale factories, further improvements are still required in the color of the fruiting bodies, and the length of the stipe of this *O. apalosarca* variety.

Cap size, cap color, and stipe length are crucial morphological characteristics of the fruiting bodies of edible mushrooms. In the industrial development of edible mushrooms, small white cap with long stipe cultivars have a dominant position in the market and possess significant commercial value, such as *F. velutipes* ans *H. marmoreus*. Improving the cap size, color, and stipe length of edible mushrooms is particularly important for their industrial cultivation. Therefore, understanding the genetic regulatory mechanisms of cap development and stipe elongating are important for the practical application of strain optimization. Light is crucial for cap differentiation, coloration and stipe elongation in basidiomycetous mushroom-forming fungi (Yang et al., 2009; Sakamoto, 2018; Feng et al., 2023). *Coprinopsis cinerea* fruiting bodies formed in complete darkness had tiny caps (Terashima et al., 2005). Cap differentiation can be observed at an unduly early stage of fruiting body development in *F. velutipes* (Sakamoto et al., 2007), and the fruiting body of *Polyporus arcularius* (Kitamoto et al., 1974) induced by light and grown in the dark displayed a long stipe without a differentiated pileus on its apex In the study of *F. velutipes*, light affects stipe elongation and thickening. The stipe elongated faster in the dark than in light. When a light-exposed fruiting body forms in the light, stipe elongation is immediately suppressed, and the stipe thickens (Sakamoto et al., 2004). By altering the duration, intensity, and wavelength of light, the color, and growth characteristics of mushrooms can be improved. For example, the fruiting bodies of *F. velutipes* exposed to green and blue light exhibited deeper colors than those grown in darkness or under red light, and the color deepened further after 48 h of blue light exposure compared with 24 h exposure (Im et al., 2024). However, the effects of light on the fruiting bodies of *O. apalosarca* and the mechanisms underlying the formation of key traits remain largely unknown.

Whole-genome analysis is a useful method for strain classification and obtaining genetic information regarding the molecular mechanisms of fungal development and breeding. However, no genetic reports on *O. apalosarca* are currently available. Even within the genus *Oudemansiella*, there is only one report on the genome of *O. raphanipes*. This has significantly hindered genetic breeding research on *Oudemansiella*, species. This study presents the assembly and annotation of the first genome of *O. apalosarca*. It examined the impact of light on cap coloration and stipe elongation. Through integrative transcriptomic analysis, key pathways and associated genes influencing cap color formation and stipe elongation were identified. These findings provide a theoretical framework and data support for further investigation of the formation mechanisms of edible mushroom fruiting bodies, enhancement of *O. apalosarca* varietal traits, and adaptation to industrial production.

## 2 Materials and methods

### 2.1 Strains and protoplast mononuclearization

The strain of *O. aparlosarca* BIPP21200004 used in this study was collected from Dadugang, Yunnan Province, China, in 2011 and preserved at the Beijing Edible Fungi Germplasm Resource Center, Beijing Academy of Agriculture and Forestry (Rebecca et al., 2021). The strains were cultured on potato dextrose agar (PDA) medium at 25°C for 7 days, then transfer the strains to PDB liquid medium cultured for 5 days. We used 2% Lywallzyme (Guangdong Institute of Microbiology, Guangzhou, China) to digest the cell walls of heterokaryotic mycelium and obtained protoplasts following the method described in a previous study (Zhao and Chang, 1993). The protoplasts were diluted and revived on cell wall regeneration medium (1% malt extract, 0.4% glucose, 0.4% yeast extract, 1.5% agar and 0.6 M mannitol) to form hyphae and colonies. Through antagonistic reactions, microscopic observation, internal transcribed spacer (ITS) region identification, and mushrooming experiments, the recovery strains were comprehensively judged to be homokaryotic or heterokaryotic. The homokaryotic strain A6 was obtained using this method. The mycelia of A6 were collected by centrifugation, washed with 0.1 M phosphate-buffered saline (PBS), and frozen in liquid nitrogen. High-quality genomic DNA was extracted from mycelia using the cetyltrimethylammonium bromide (CTAB) method. RNase A (Leagene, Beijing, China) in 10 µg/mL was used to remove RNA from the samples.

### 2.2 Genome assembly and annotation

The extracted DNA molecules were sequenced with both the Illumina NovaSeq6000 (Illumina Inc., San Diego, CA, USA), and the PacBio Sequel (Pacific Biosciences of California, Menlo Park, CA, USA) platform at Novo-gene Bioinformatics Technology Co., Ltd. (Beijing, China). The short reads from the Illumina platform were quality-filtered using high-throughput quality control (HTQC) following by removed low-quality bases (quality value ≤20) and overlap with adapters of >15 bp reads, respectively (Yang et al., 2013). We conducted a preliminary assembly based on Illumina data and initially assessed the genome size, heterozygosity, and repeat sequence information of the samples using K-mer analysis. We generated the 15-mer occurrence distribution of sequencing reads from short libraries for A6 strain. SMRTbell libraries were sequenced on a PacBio Sequel system. The sequencing reads were processed in four stages: subread correction, assembly of corrected reads, single-base correction, and linking contigs to scaffolds and gap filling (Kim et al., 2014; Badouin et al., 2015; Faino et al., 2015; Sit et al., 2015; Tsuji et al., 2015). The sub-reads were corrected using Proovread (v2.12). Corrected reads were assembled using Falcon (v0.3.0) and Celera Assembler (v8.3), and the optimal assembly was selected. Single-base errors in the assembly were corrected using Illumina HiSeq data and GATK (v1.6-13). The contig was linked to the scaffold with long inset-size pair-end reads using SSPACE_Basic_v2.0; then, the gap was filled using the software pbjelly2 (15.8.24).

Homology-based and *de novo* predictions were combined to identify the repeat content in the A6 genome. Homology prediction was performed genewise (2.20) (Birney, 2004), and SNAP (version 2010-07-28) (Johnson et al., 2008) and Augustus (3.2.1) (Stanke et al., 2008) were used to predict genes. The RNA-seq data obtained from RNA extraction of mycelium cultivated with PDA for gene prediction. Finally, we integrated the prediction results of various software packages using EVM (1.1.1). Gene functions were inferred according to the best match of the alignments to the National Center for Biotechnology Information (NCBI), Non Redundant (NR), InterPro (Alex et al., 2015), and Swiss Prot (Boeckmann and Research, B. J. N. A, 2003) protein databases using Basic Local Alignment Search Tool for Proteins (BLASTP) (Camacho et al., [[NoYear]]) and the Kyoto Encyclopedia of Genes and Genomes (KEGG) database (Minoru et al., 2012) with an E value threshold of 1 × 10 ^−5^. The Gene Ontology (GO) (Ashburner et al., 2000) ID for each gene was obtained from Blast2GO (Conesa and Götz, 2008). We found rRNAs by comparison with the rRNA database or prediction with RNAmmer (1.2) (Karin et al., 2007). Using tRNAscan (1.3.1), the area of the tRNA and secondary structure were predicted (Lowe and Eddy, 2019). Using the Infernal database compared with the Rfam (9.1) database, we obtained sRNAs (Gardner et al., 2008). The transposon sequence was identified by aligning the assembly with a known transposon sequence database, using the *de novo* method. The RepeatProteinMask search uses a transposable element (TEs) database as a query library. *De novo* repeat prediction involved using the buildXDF Database to construct a database from the assembly sequences, creating transposon models with RepeatModeler (open-1.0.11), and subsequently identifying transposons with the established models using RepeatMasker (4-0-6) (Chen, 2004). Tandem repeat sequences were predicted using the Tandem Repeat Finder (TRF) software (4.04) (Gary, 1999).

### 2.3 Treatment under different light conditions

**Figure 1:**
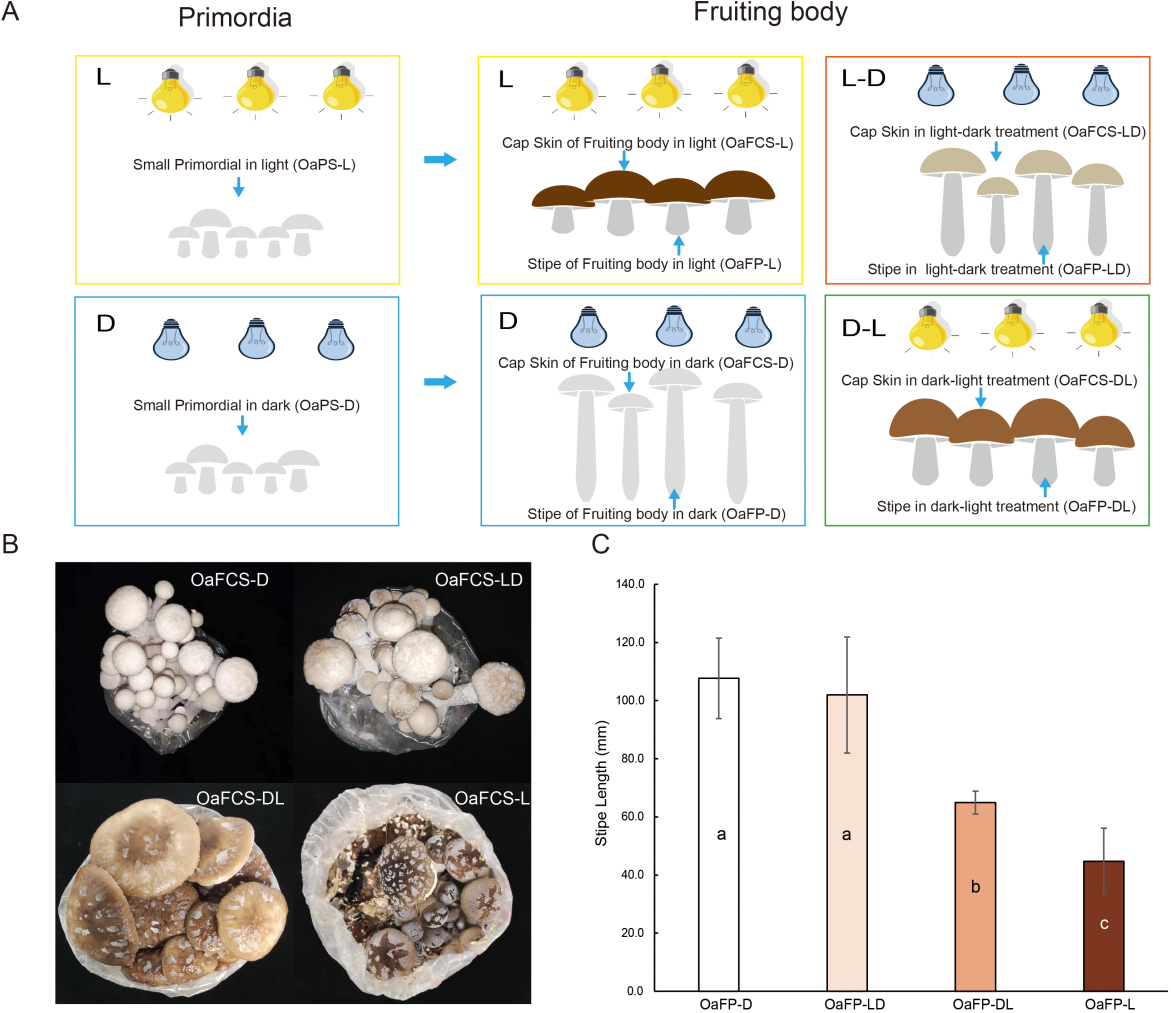
Different light treatments and phenotypes of *Oudemansiella apalosarca* fruiting body. **(A)** Representation of light and dark treatment at different developmental stages. The left side shows light or dark treatment during the primordium stage. Light and dark treatment during the fruiting body development stage are shown on the right. **(B)** Different colors of the mushroom cap after different light treatments. **(C)** Length of mushroom stipes after different light treatments. Different lowercase letters indicate significant differences in stipe length when P<0.05. For an explanation of the abbreviations for the treatment methods, refer to **Supplementary Table S8**.

The BIPP21200004 strain was initially cultivated on PDA medium until the mycelia reached three-quarters of the size in the Petri dish. It was then transferred to a culture bottle (480 ml) with 500 g weight comprising 60% cottonseed hulls, 38% bran, 2% lime, which had been autoclaved at 121°C for 120 min with a moisture content of 60%, and incubated in the dark. Upon full colonization of the bottles (around 20 days), the mycelia were packed into cornerfold plastic bags (22×33 cm) with 1.25 kg same compose of cultural bottles, sterilized, and inoculated with the strain. Incubation continued at 23–25°C until full substrate colonization (23–25 days), after which the colonized substrate was transferred to a controlled intelligent mushroom incubator (humidity 90%, temperature 20°C, natural ventilation) for fruiting. After 30 d of cultivation, the primordia were formed. During the primordial stage, half of the mushroom bags were placed in an incandescent light illumination for 2 d (**Figure 1A**). After the light treatment, the mushroom bags containing primordia were split, with one half subjected to continuous light cultivation and the other half cultivated in a dark room until the fruiting bodies matured (**Figure 1A**). The mushroom bags that had formed primordia in the dark room underwent a similar treatment, where after splitting, one half of the mushroom stems continued to be cultivated in the dark and the other half was subjected to light cultivation (**Figure 1A**). About 6 days after the primordia formation, the fruiting bodies developed and matured. Following the maturation of the fruiting bodies, samples from caps and stipes were collected under four conditions (light, dark, light-to-dark, and dark-to-light cultivations), with three biological replicates per condition. Ten periods and 30 samples were selected for transcriptome sequencing. The cap that removed the gills and stipes that removed about 1–2 cm from connect part with cap, was cut into small pieces and place them separately into centrifuge tubes, respectively. Tubes were immediately frozen in liquid nitrogen and stored at −80°C for future analyses.

### 2.4 Transcriptome sequencing and analysis

RNA was extracted from the samples, using TRIZOL^®^ Reagent (Invitrogen, USA). The RNA quality and integrity were evaluated using a NanoDrop 2000 spectrophotometer (Thermo Fisher Scientific, USA) and an Agilent 2100 bioanalyzer (Agilent Technologies, USA), respectively. Library construction and RNA sequencing were performed by Frasergen Bioinformatics Co. Ltd. (Wuhan, China). cDNA libraries were prepared using a NEBNext^®^ Ultra™ RNA Library Prep Kit for Illumina^®^ (NEB, USA). Subsequently, the libraries were evaluated for qualification and pooled based on the effective concentration and sequencing requirements. The pooled libraries were then sequenced on an Illumina platform with 150 bp paired-end reads.

Quality control of the raw data was performed using the FastQC plugin of TBTools v2.154 (Simon, 2010; Chen et al., 2023). After removing low-quality paired reads and adaptor contamination using the Trimmomatic plugin, the clean reads were spliced and aligned to the reference A6 genome using the Hisat2 plugin. Gene expression levels were measured based on fragments per kilobase of transcript per million mapped fragments (FPKM), and the expression matrix was obtained using the StringTie quantification plugin. Differentially expressed genes (DEGs) were screened using DESeq2 (|log2 (fold change [FC])| > 1, FDR < 0.05). Gene set enrichment analysis (GSEA) is a statistical method used to evaluate whether predefined gene sets exhibit significant differences under different biological conditions. In this study, GSEA was used to analyze the enrichment of GO terms among DEGs. GO terms with P <0.05 and FDR <25% were considered to be significantly enriched. KEGG and GO enrichment analyses were performed using the OmicShare tools (www.omicshare.com/tools). Significantly enriched pathways and GO terms among rearranged genes were compared to syntenic genomes by the hypergeometric test. The calculated p-value was adjusted through FDR correction, with FDR < 0.05 as a threshold. Pathways and GO terms meeting this condition were defined as significantly enriched. KEGG enrichment analysis was used to identify the DEGs involved in the major biochemical metabolic pathways and signal transduction pathways (P <0.05).

### 2.5 Gene family analysis and quantitative real-time polymerase chain reaction verification

Using BLASTP analysis, the tyrosinase genes were identified in the A6 genome. We downloaded the tyrosinase protein sequences of 19 fungi from the NCBI (**Supplementary Table S1**), performed multiple sequence alignment using MUSCLE, and constructed a phylogenetic tree using the maximum likelihood method. MEME (5.5.7) (Bailey et al., 2015) was used for motif prediction and Batch CD-Search (Wang J. et al., 2023) to predict conserved domains in gene families. Images were visualized using a combination of TBtools (v2.154) and Adobe Illustrator CS6. Real-time qPCR of DEGs was performed using TB Green ^®^ Premix Ex Taq™ II (Tli RNaseH Plus, TaKaRa, Japan) on an ABI 7500 real-time PCR system (Applied Biosystems, USA) (Yan et al., 2021). The primer information is provided in **Supplementary Table S2**.

## 3 Results

### 3.1 Characterization of the *O. aparlosarca* genome

Previous research indicates that the mycelial cells of *O. aparlosarca* exhibit binucleate and multinucleate forms, with clamp connections being difficult to observe (Terashima et al., 2005). Thus, determining whether protoplast-regenerated strains are homokaryotic based solely on microscopic observation of clamp connections is challenging. Initial antagonistic tests were conducted on the regenerated strains, with those showing antagonism with the parental strain subjected to further screening. Optical microscopy was then employed to observe the presence or absence of clamp connections in the selected strains. Strains lacking clamp connections underwent another round of selection. The ITS region of the selected strains was amplified using PCR with ITS4 and ITS5 primers and sequenced using Sanger sequencing. For heterokaryotic parental strains, bases following CTCTCTCTC exhibited G/A peaks; presence of solely A or G indicated potential homokaryotic status (**Supplementary Figure S1A**). Fruiting tests were subsequently conducted to verify the fruiting ability of the candidate strains. Strains unable to form fruiting bodies independently were considered homokaryotic. Finally, a homokaryotic protoplast-regenerated strain A6 of *O. aparlosarca* was obtained. High-throughput sequencing survey analysis revealed that the heterozygosity of strain A6 was merely 0.4%, indicating extremely low heterozygosity (**Supplementary Figure S1B**). These results further confirmed that strain A6 was homokaryotic.

**Figure 2:**
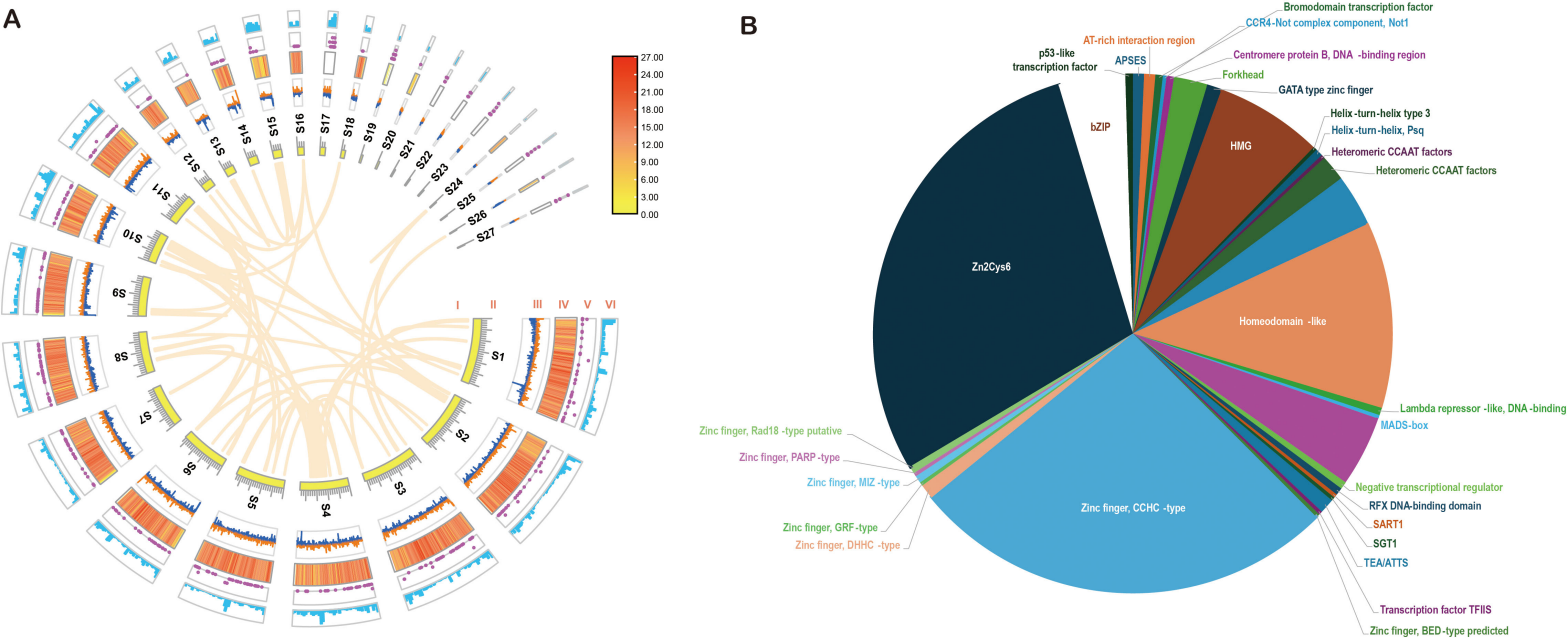
Genome circos image and transcription factor (TF) of *Oudemansiella apalosarca*. **(A)** Circos image of genome. I: Collinearity relationship between genomes; II: scaffold; III: GC stew; IV: protein cording genes; V: non-cording RNA; VI: repeats. **(B)** TF types of the *O. apalosarca* genome.

The genome of homokaryon A6 of *O. aparlosarca* strain BIPP21200004 was sequenced using the Illumina HiSeq and PacBio Sequel platforms. Analysis of the assembled scaffold and genome is presented in **Supplementary Table S3**. The single nucleotide error rate was 0.03%, after subread correction, 11,510,535,780 bp (216X) were used for assembly. We assembled a total length of 53.13 Mb from the A6 genome with 27 scaffolds (including 28 contigs) and a scaffold N50 of 4.31 Mb, scaffold N90 = 1.07 Mb. The maximum scaffold length is 6.10 Mb. Only a single gap exists in the entire genome. The GC content of the assembled scaffold was 49.5%. The GC stews exhibited various characteristics in different scaffolds (**Figure 2A**). The benchmarking universal single-copy orthologs (BUSCOs, version 5.5.0) gene set of fungi_odb10 was used to assess assembly qualities. A total of 758 BUSCOs were determined in the A6 genome assemblies, and the complete BUSCO rate was 98.4% (**Supplementary Table S3**).

Based on homologous proteins, transcriptional data, and *de novo* prediction, the *O. aparlosarca* genome includes 14,650 coding protein genes, which is 51% of the entire genome, and the average length of protein-coding genes was 1839.21 bp (**Supplementary Table S4**). The genes were annotated using six databases: GO, IPR, KEGG, KOG, NR, and SWISSPROT, and 75.69% of the genes can be annotated to the six databases (**Supplementary Table S4**). The GO functional annotation results indicate that 3,401 genes were involved in metabolic processes, 3,928 genes were associated with binding functions, and 3,451 genes exhibited catalytic activity (**Supplementary Figure S2A**). The KEGG annotation results revealed that the metabolic pathways of cell growth and death comprised 214 genes, carbon metabolism included 390 genes, amino acid metabolism encompassed 468 genes, and secondary metabolism consisted of 77 genes (**Supplementary Figure S2B**). Transcription factors (TFs) are involved in the regulation of important life activities within organisms. The analysis results showed that A6 has 33 types of TFs, with a total of 432 genes, among which Zn2Cys6 type TFs account for the largest cumulative proportion of 125 (**Figure 2B**). Different scaffold genes had different distribution densities, and no coding genes were predicted on scaffolds 17, 21, 22, 23, 25, and 27. However, a relatively dense distribution of ncRNAs and repetitive sequences was observed on the scaffolds (**Figure 2A**). Collinearity existed within the genome, indicating the presence of multiple copies of genes on different scaffolds (**Figure 2A**). The A6 strain genome contained five annotated types of non-coding RNAs: tRNA, rRNA, sRNA, snRNA, and miRNA (**Supplementary Table S5**). Repetitive sequences represent approximately 14.31 of the A6 genome (**Supplementary Table S6**). Long-terminal repeats (LTR) comprised 6.09% of the A6 genome.

**Figure 3:**
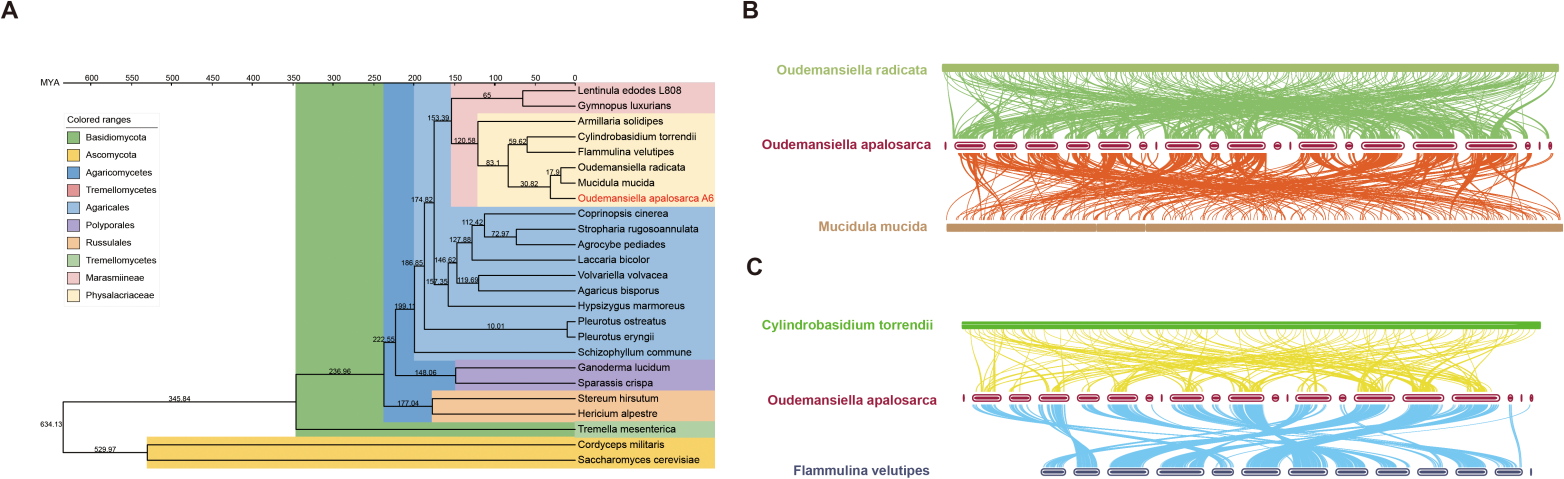
Genome evolutionary and comparative analysis of *Oudemansiella apalosarca*. **(A)** A phylogenetic tree was constructed using the maximum likelihood method based on 193 single-copy orthologous genes from *O. aparlosarca* and 24 other fungal species (22 basidiomycetes and two ascomycetes). Divergence times are represented textually at internal nodes, with the 95% highest posterior density presented in millions of years ago (MYA) on the x-axis. **(B)** Genome collinearity among *O. aparlosarca*, *O. radicata*, and *Mucidula mucida*. **(C)** Genome collinearity among *O. aparlosarca, Cylindrobasidium torrendii*, and *Flammulina velutipes*.

Phylogenetic analyses were conducted to investigate evolutionary relations between *O. aparlosarca* and 25 other fungal species (23 basidiomycetes and 2 ascomycetes, **Supplementary Table S7**). Similarly, 193 single-copy orthologous genes were observed and used for phylogenetic reconstruction and species divergence time estimation (**Figure 3A**). The results showed that basidiomycetes and ascomycetes could be well distinguished and that species of the same family could form a cluster, such as the family Physalacriaceae. *Oudemansiella aparlosarca* clustered with *O. radicata* and *Mucidula mucida*, diverging around 40.7 million years ago from the two species. Its divergence time from *Cylindrobasidium torrendii* and *F. velutipes*, which also belong to Physalacriaceae, is over 100 million years. Similarly, the genomic collinearity analysis indicated that *O. aparlosarca* shares more collinearity with *O. radicata* and *M. mucida*, indicating a closer relationship (**Figures 3B, C**).

### 3.2 Effects of different light treatments on the phenotype of *O. aparlosarca*

We conducted light and dark treatments on the primordia formation and fruiting body developmental stages of *O. aparlosarca* strain BIPP21200004 to investigate the effect of light on cap color and stipe elongation. These results indicate that *O. aparlosarca* is sensitive to light. In the fruiting experiments, both the primordia and fruiting bodies were grown in the dark and exhibited white caps (**Figures 1A, B**, **Supplementary Table S8**), whereas those cultivated under continuous light conditions had dark brown caps (**Figures 1A, B**, **Supplementary Table S8**). When primordia grown in light were transferred to a dark environment until fruiting body maturation, the cap color remained light brown (**Figures 1A, B**, **Supplementary Table S8**). Conversely, when primordia grown in the dark were shifted to light until maturity, the cap color changed from white to brown (**Figures 1A, B**, **Supplementary Table S8**). In length of stipe, the stipe length of the strain cultivated continuously in the dark during the fruiting body development stage was significantly higher than that of stipes cultivated in the light (**Figure 1C**). The stipe length of the samples subjected to light treatment during the primordium formation stage was the lowest, and there were significant differences compared to the other treatments (**Figure 1C**).

### 3.3 Comparison between cap and stipe transcriptome analysis during different light treatments

A total of 1.79 Gb clean reads with Q20 values greater than 94% were obtained, and the average total read mapping was 85.46% (**Supplementary Table S9**). The Pearson correlation coefficient between the biological replicates indicated the reliability and validity of the RNA-seq data (**Supplementary Figure S3**). Correlations between samples from the primordium, cap, and stipe stages were weaker. To further analyze the correlations between samples, principal component analysis (PCA) was applied to the gene count data of each sample. The PCA results indicated that, compared to the light treatment, the differences among samples at different growth stages were greater (**Supplementary Figure S4**). In total, 30744 DEGs were identified in the seven categories understand the mechanisms through which light affects the growth and development of *O. aparlosarca*. The DEG categories of OaFCS_L *vs*. OaFCS_D (2522 upregulated and 2593 downregulated genes), OaFCS_L *vs*. OaFCS_DL (3319 upregulated and 462 downregulated genes), OaFCS_L *vs*. OaFCS_LD (2293 upregulated and 2403 downregulated genes), OaFP_L *vs*. OaFP_D (2048 upregulated and 2353 downregulated genes), OaFP_L *vs*. OaFP_DL (2239 upregulated and 2556 downregulated genes), OaFP_L *vs*. OaFP_LD (2228 upregulated and 2606 downregulated genes), and OaPS_L *vs*. OaPS_D (76 upregulated and 46 downregulated genes) were compared to (**Supplementary Figure S5**). In the primordium stage, the number of DEGs between light and dark treatment was fewest. In the cap and stipe, compared with the samples placed in the light, the number of downregulated DEGs was higher than that of the upregulated ones (**Supplementary Figure S5**).

**Figure 4:**
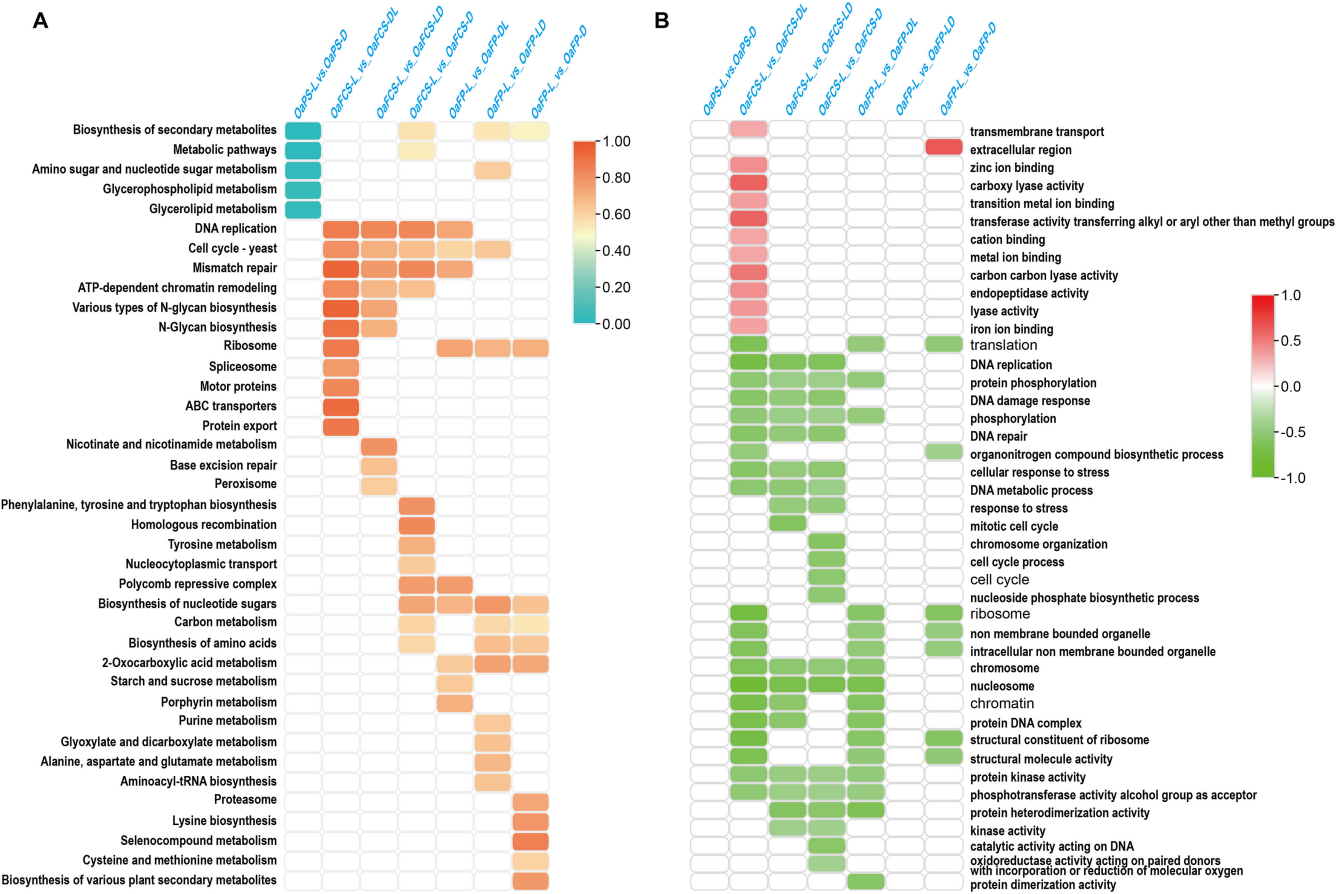
Kyoto Encyclopedia of Genes and Genomes (KEGG) enrichment and Gene Ontology (GO) gene set enrichment analysis (GSEA) of differentially expressed genes (DEGs) between different light treatments. **(A)** KEGG enrichment of DEGs between different light treatments in the primordium, cap, and stipe. The vertical coordinate represents the gene ratio in different KEGG pathways. The larger the gene ratio, the deeper the red coloration. The figure shows the KEGG pathways with P<0.05. **(B)** Selected GO terms with statistically significant and concordant differences between different light treatments, as revealed through GSEA. The vertical axis represents the enrichment score of different GO terms, with red indicating positive values and green indicating negative values. Red signifies that the expression levels of most genes in that GO term increased compared with those in the fully illuminated group. Green indicates that the expression levels of most genes were decreased compared with those in the fully illuminated treatment group. The image shows gene sets with FDR < 25% and ranked in the top 20.

**Figure 5:**
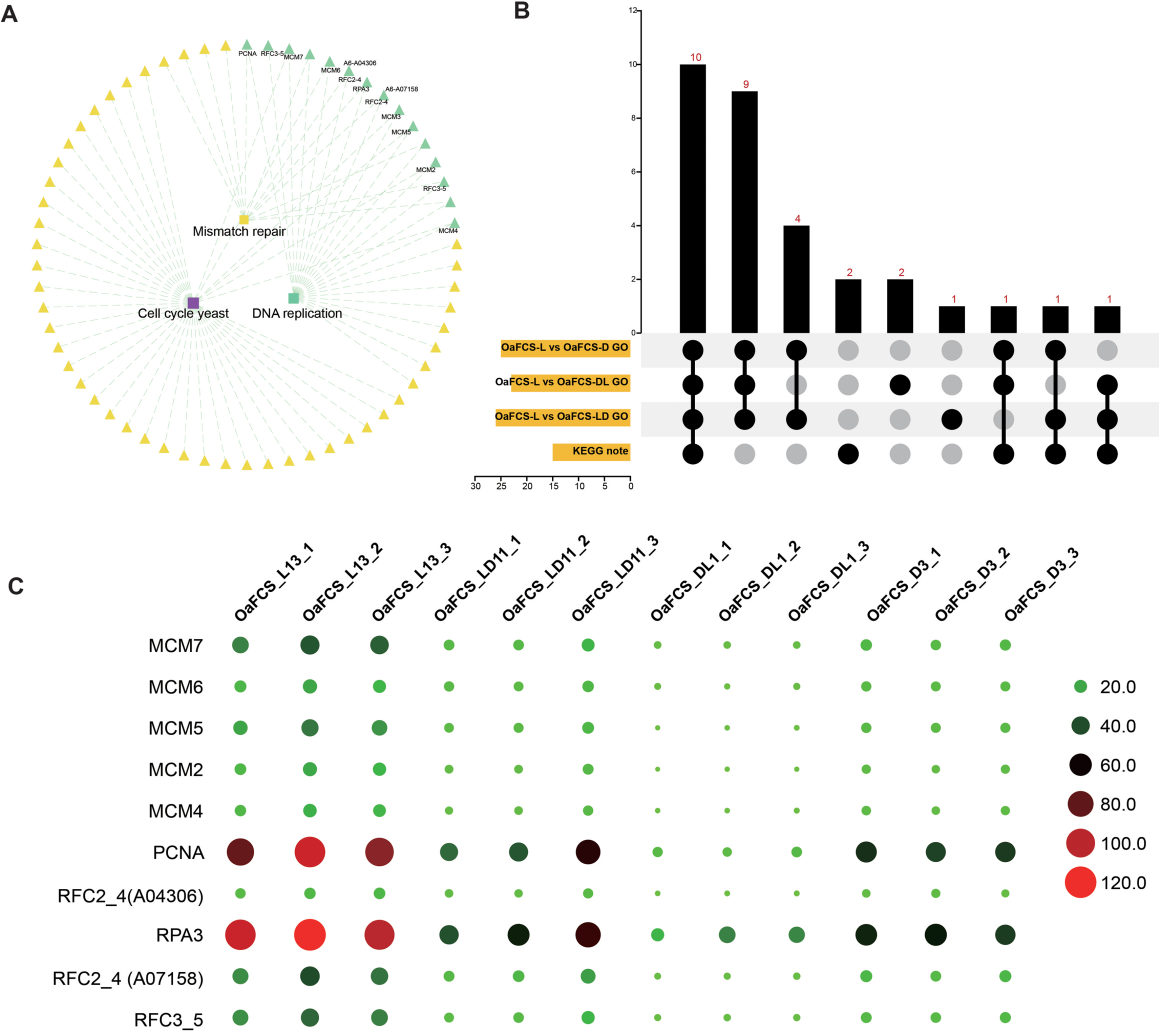
Common DEG expression patterns of the DNA replication term in KEGG and GSEA. **(A)** Net image of commonly enriched KEGG pathways in the cap surface. Note genes are represented in green. **(B)** Upset plot of DEGs in the DNA replication term. **(C)** Common DEG expression patterns in the cap surface of KEGG nodes and GSEA contributing genes. The larger and more reddish the dot, the higher the expression level.

We conducted KEGG and GSEA enrichments of the DEGs. All significantly enriched KEGG pathways (P < 0.05, **Figure 4A**) and the top 20 significantly enriched GO terms (FDR <25%) identified through GSEA (**Figure 4B**) were selected to analyze the effects of light on primordium, cap, and stipe developments. Compared with those placed in the light, the DEGs in the darkroom cultivation were primarily enriched in five metabolic pathways: the biosynthesis of secondary metabolites, metabolic pathways, amino sugar and nucleotide sugar metabolisms, glycerophospholipid metabolism, and glycerolipid metabolism (**Figure 4A**). Based on the GSEA, there were no annotations for the significantly enriched GO gene sets in the primordial stage (**Figure 4B**). GSEA indicated that, except for the significant positive enrichment of DEGs between OaFCS_L and OaFCS_DL, other comparisons showed significant negative enrichments. This implies that, compared with those in the fully illuminated group, the genes significantly enriched in the GO terms exhibited downregulated expression levels in the mushroom cap and stipe (**Figure 4B**). In the samples of the cap surface, compared with those under light cultivation, all DEGs from the samples cultivated in the dark were enriched in the DNA replication term, as revealed by KEGG and GSEA (**Figures 5A, B**). Furthermore, we analyzed the expression patterns of genes related to DNA replication. The GSEA indicated that DNA replication was significantly negatively enriched among the DEGs in OaFCS_L *vs*. OaFCS_D, OaFCS_L *vs*. OaFCS_DL, and OaFCS_L *vs*. OaFCS_LD (**Supplementary Figure S6**). The KEGG network diagram showed that the DNA replication pathway is correlated with the co-enriched KEGG pathways of mismatch repair and cell cycle (**Figure 4A**). We performed an association analysis between the note genes in the aforementioned KEGG pathways and the core enrichment genes from GSEA, revealing 10 common genes (**Figure 5B**). These 10 common genes contain five mini chromosome maintenance (MCM) family protein, three replication factor C (RFC), and proliferating cell nuclear antigen (PCNA). The expression patterns of these 10 common genes were consistent with the GSEA, showing significantly lower expression in the caps of fungi cultivated in dark environments than in those cultivated under complete light conditions (**Figure 5C**).

In the stipe, the ribosome-related terms were co-enriched, based on KEGG and GSEA. The DEGs in the GSEA for OaFCS_L *vs*. OaFCS_DL showed no significant enrichment of GO terms. The core gene sets of the ribosome-related terms in OaFCS_L *vs*. OaFCS_D and OaFCS_L *vs*. OaFCS_LD were 88% and 91%, respectively, indicating that ribosome-related genes exhibited significantly downregulated expression after dark treatment (**Supplementary Figure S7AB**). The expression patterns of the 43 common DEGs revealed by KEGG under different light treatments in the fruiting body also confirmed this (**Supplementary Figure S7C**). Additionally, in the commonly enriched KEGG pathways of the stipe, pathways such as nucleotide sugar biosynthesis, carbon metabolism, amino acid biosynthesis, and 2-oxocarboxylic acid metabolism showed good correlations (**Supplementary Figure S7D**). Eight associated node genes were identified, of which four were upregulated, and four were downregulated after darkroom cultivation (**Supplementary Figure S7E**). Additionally, we analyzed the expression of nine significant genes which associated with fungal cell formation in the stipe, and exhibited high expression in dark treatment (**Supplementary Figure S7E**).

### 3.4 Expression of tyrosinase synthesis exhibits a positive correlation with cap color

**Figure 6:**
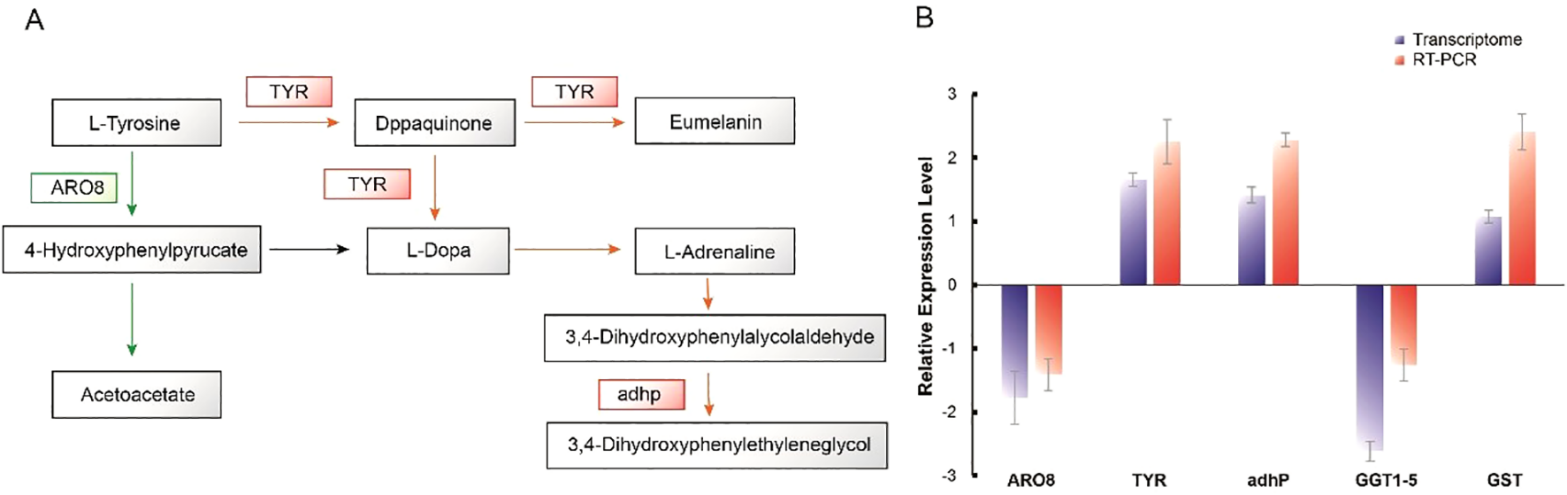
Synthesis pathway and key gene expression of pigments in the cap of *Oudemansiella apalosarca*. **(A)** Tyrosine metabolism pathway. **(B)** RT-qPCR validation of pigments synthesis key gene expression.

In the KEGG pathways that were significantly enriched in the caps of fungi cultivated in complete darkness and full light conditions, phenylalanine, tyrosine, and tryptophan biosynthesis and tyrosine metabolism were notably enriched (**Figure 4A**). These two pathways may be related to the regulation of cap color changes. Tyrosinase participates in the production of dopaquinone from tyrosine in eumelanin biosynthesis, and in the synthesis of L-DOPA from tyrosine in catecholamine biosynthesis (**Figure 6A**). These steps play crucial rate-limiting roles in eumelanin and catecholamine synthesis. Therefore, tyrosinase may be the key factor leading to the deepening of the cap color in *O. aparlosarca*.

**Figure 7:**
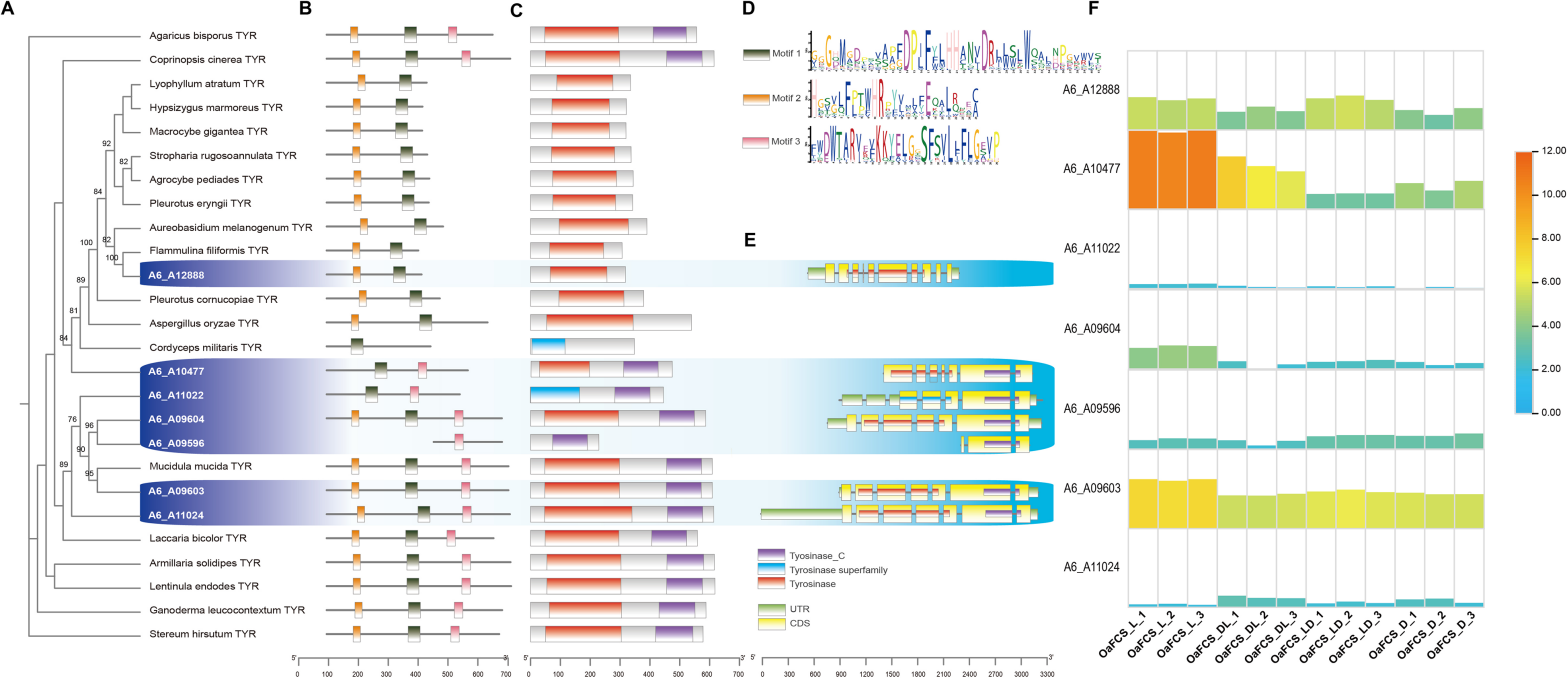
Tyrosinase gene family analysis. **(A)** Phylogenetic tree of tyrosinase proteins from 19 other fungal species and seven *O. apalosarca* tyrosinase proteins. **(B)** Motif structures of tyrosinase proteins. **(C)** Conserved domains of tyrosinase proteins. **(D)** Motif of tyrosinase. **(E)** Gene structures of *O. apalosarca* tyrosinase. **(F)** Expression patterns of seven tyrosinase-encoding genes of *O. apalosarca*. The more the color shifts toward red, the higher the expression level.

The gene encoding tyrosinase in *O. aparlosarca* is a multicopy gene. Seven related tyrosinase (TYR) genes were identified in the A6 genome (**Figure 7A**). The results indicated that six TYR proteins of *O. aparlosarca* have different origins. The TYR proteins A6–10477 and A6–12888 belong to evolutionary branches that are different from those of the other five *O. aparlosarca* TYR proteins, with A6–12888 being consistent with the TYR protein sequence of *Flammulina filiformis* (**Figure 7A**). The three proteins A6-11022, A6-09604, and A6–09596 are closely related. A6–09603 is closely related to the TYR protein of *Mucldula muclade* (**Figure 7A**). Using MEME to analyze the motif structures of the 26 proteins, three motifs were identified (**Figures 7B, D**). Among these, 11 proteins possessed three complete motif structures (**Figure 7B**). A6_A12888 contained only motif 2, whereas A6_A09604 contained only motifs 1 and 3 (**Figure 7B**). Using CD-search to analyze the conserved domains, three domains were identified, namely tyrosinase C, tyrosinase superfamily, and tyrosinase (**Figure 7C**). Except for *A6_A12888*, other tyrosinase-encoding genes of *O. aparlosarca* contained a tyrosinase C domain. Except for *A6_A11022*, other tyrosinase-encoding genes of *O. aparlosarca* contained a tyrosinase domain. In A6_A11022, the tyrosinase domain was annotated as a tyrosinase superfamily domain. In addition, there were differences in the gene structure among the seven tyrosinase-encoding genes (**Figure 7B**). The CDS region structures of A6_A09603, A6_A09604, and A6_A11–24 genes were relatively similar (**Figure 7E**). The expression patterns of these seven tyrosinase-encoding genes in cap epidermis samples under different light treatments were analyzed (**Figure 7F**). The results showed that, except for *A6_A09596* and *A6_A11024*, the genes were upregulated in the cap epidermis under full-light culture conditions. Among these, the highest expression was observed for *A6_A10477*, followed by *A6_A09603*. The expression levels of these two genes were significantly higher than those of other tyrosinase-encoding genes in all samples.

Aromatic amino acid transaminase (ARO8) is involved in the tyrosine degradation pathway, primarily catalyzing the formation of 4-hydroxyphenylpyruvate from tyrosine. Compared with dark-grown caps, the expression level of ARO8 was significantly reduced (**Figure 6B**). Alcohol dehydrogenase (adhP), a member of the tyrosine metabolism pathway, uses nicotinamide adenine dinucleotide (NAD) as a coenzyme to catalyze the reversible reaction between primary alcohols and aldehydes. Numerous studies have shown that adhP primarily participates in ethanol fermentation, with its primary function being the conversion of acetaldehyde to ethanol, the generation of NAD+, and limited ATP, which is then converted to NADPH, finally producing DHN-melanin. The expression level of adhP significantly increased on the light-treated cap surface (**Figure 6B**). In addition, the glutathione metabolism pathway was enriched in the DEGs of *O. apalosarca* caps exposed to light and in those cultured in the dark. Notably, glutathione is also involved in pheomelanin biosynthesis (Benathan et al., 1999). Glutathione-S-transferase (GST) is a key enzyme in the glutathione conjugation reaction, catalyzing the initial step of the glutathione conjugation reaction. When cysteine is present in the cell, dicarbonyl immediately binds to cysteine to form cysteamine dopa, which then produces pheomelanin through various enzymatic reactions (Ito and Wakamatsu, 2003). Pheomelanin accumulation may cause changes in the color of the fruiting bodies of *Auricularia* (Li et al., 2021). Compared to the dark-cultured fruiting bodies of *O. apalosarca*, the GST gene was upregulated. Finally, we performed RT-qPCR validation of the expression levels of tyrosinase-encoding genes A6_A09603, ARO8, adhP, γ-glutamylcysteine (GGT1_5), and glutathione S-transferase (GST) in cap surface samples in complete light and darkness. The RT-qPCR validation results were consistent with those of the transcriptome analysis (**Figure 6B**).

## 4 Discussion

### 4.1 Genome evolutionary of *O. apalosarca*

The scientific naming of the *Oudemansiella* genus is controversial in the scientific community. In the early 1990s and 2000s, Yang (2000) described and documented nearly 20 species of the genus *Oudemansiella* in the southwestern region. In 2009, Yang et al. (Yang et al., 2009) collaborated with researchers from the United States and Germany to revise the taxonomy of the narrow-sense *Oudemansiella*, proposing a new system of *Oudemansiella* comprising four groups: *Oudemansiella*, *Mucidula*, *Dactylosporina*, and *Radicatae*. Hao et al. (Hao et al., 2016) classified it as the genus *Oudemansiella* in 2016 based on the combined ITS and nrLSU; however, it has also been classified as a species of other genera, primarily *Hymenopellis* (Chen, 2004). The genome of the genu*s Oudemansiella* is named *Hymenopellis* in the NCBI. There are usually inconsistencies in the characteristics used to differentiate closely related species, making it challenging to compare fungal taxa defined by different species identification standards. Classifying and identifying fungi based on genomic sequences is an effective approach (Xu, 2020). Studies on the genomics of *O. apalosarca* are limited. There were only four genomic information entries for the *Oudemansiella* genus in the NCBI, with two belonging to *O. radicata* and two assigned to *O. raphanipes*. Only the *O. radicata* IJFM A160 strain has gene annotation information. In this study, we assembled and annotated the first genome of *O. apalosarca* at the scaffold level, with the highest scaffold N50 and BUSCO value among *Oudemansiella* genus genomes (**Supplementary Table S3**). This lays the foundation for subsequent systematic studies of the evolutionary patterns of the *Oudemansiella* genus.

The construction of a phylogenetic tree using whole-genome single-copy genes revealed that *O. apalosarca* is closely related to the *O. radicata* IJFM A160 strain and *M. mucida*. Furthermore, the *O. radicata* IJFM A160 strain and *M. mucida* occupy the same evolutionary branch. This is consistent with the results of the phylogenetic tree construction in a previous study using the genome of *O. raphanipes* CGG-A-s1 (Zhu et al., 2023). The study also indicated that there are significant differences between the genomes of *O. raphanipes* CGG-A-s1 and those of *O. radicata* MG139 and *O. radicata* IJFM A160 (Zhu et al., 2023). We used the genome fasta file and performed comparative analysis using MUMmer and BLAST, discovering that there is no synteny between the A6 genome sequence and the three genomes, including the IJFM A160 strain of *O. radicata*. The A6 genome showed synteny with the CGG-A-s1 strain of *O. raphanipes* only on scaffold 3 (**Supplementary Figure S8**). This may be owing to the incomplete assembly of the other three genomes, which have a higher number of contigs and lower homology of DNA sequences, leading to poor synteny. In addition, we conducted collinearity analysis through MCScan using protein sequences and discovered a collinear relationship between the A6 genome and *O. radicata* IJFM A160. This may be because early constructed genomic strains usually originate from fruiting bodies or heterokaryotic mycelia, resulting in a higher heterozygosity rate in the genome, as seen in the *O. radicata* MG139 genome (Zhu et al., 2023). The identification of heterozygous sites in a heterozygous genome complicates comparative analyses between genomes. Moreover, the presence of repetitive sequences and high copy numbers in fungal genomes causes poor homology relationships among closely related species based on DNA sequence analysis (Mohanta and Bae, 2015). In contrast, conducting synteny analysis using protein sequences eliminates the influence of repetitive sequences between genes and the sequence differences owing to synonymous mutations among alleles. Thus, synteny relationships among closely related species can be identified. However, high-quality genomes remain crucial for studies on fungal evolution (Wang Z. et al., 2023). This is particularly true for investigating the co-evolution and horizontal transfer among alleles, which have unique advantages.

### 4.2 Light affects fungal metabolic and physiological processes

Light is a pivotal factor that affects the growth and development of edible fungi, but it may not always be necessary (Sakamoto, 2018). Fruiting body production can be induced under complete darkness in some basidiomycetes, the caps exhibit impaired development (Kües and Liu, 2000; Sakamoto et al., 2004). In this study, *O. apalosarca* formed caps in complete darkness; however, the caps were smaller than those formed under light conditions (**Figure 1B**). Our enrichment analysis supports this hypothesis. Compared with the expression in the light cultivation, core-enriched DEGs, 10 common genes from DNA replication were significantly downregulated in the dark caps (**Figure 5C**). The MCM2–7 complex, as a core component of the DNA helicase, may form a head-to-head double hexamer structure during the initiation phase of DNA replication in fungi, encapsulating double-stranded DNA and participating in the selection and activation of replication origins (Jia and Yu, 2024). RFC is a key regulatory factor in the processes of DNA replication and repair (Koh and Park, 2025). As a loading factor for PCNA, it assists DNA polymerases δ and ϵ in binding to the primer DNA template for chain elongation (Zheng et al., 2024). The differences in the expression of these core proteins indicate that under light conditions, the caps of *O. apalosarca* exhibit active DNA replication and cell cycle progression. Cell cycle regulation drives the division of cap cells, DNA replication provides the basis for genetic material, and mismatch repair ensures the accuracy of replication, leading to larger caps in light-cultured conditions than in dark-room cultures. N-glycans may influence the structure of cap cells by modifying cell wall components (Wang et al., 2025).

Under dark cultivation conditions, carbon metabolism pathways in the stipes of *O. apalosarca* were significantly enriched. Among them, the gene expression levels of citrate synthase (CS), malate dehydrogenase (ME2), isocitrate lyase, and pyruvate dehydrogenase E1 component (aceE) were significantly upregulated, indicating that the tricarboxylic acid and glyoxylic acid cycles in the stipe of *O. apalosarca* were significantly enhanced, providing substantial energy for stipe elongation under dark conditions. In addition, the pathway of nucleotide sugar biosynthesis was significantly enriched in all DEGs from fungal stipe samples after dark treatment. The inner layer of the fungal cell wall primarily comprises polysaccharides (chitin, β-1,3-glucan, α-1,3-glucan, galactosaminogalactan, and galactomannan) (Kadooka et al., 2025). These polysaccharides form a rigid skeleton through glycosidic bonds, providing mechanical strength and osmotic pressure resistance to the cell wall. In dark-cultivated *O. apalosarca*, the expression levels of UDP-glucose-1-phosphate uridylyltransferase (UGP2), UDP-glucose 4-epimerase (galE), mannose-1-phosphate guanylyltransferase (GMPP), and UDP-N-acetylgalactosamine diphosphatase (UAP1) were significantly upregulated. UGP2, galE, GMPP, and UAP1 catalyze the production of UDP-glucose (UDP-Glc), UDP-galactose (UDP-Gal), GDP-mannose (GDP-Man), UDP-N-acetylglucosamine (UDP-GlcNAc), and N-acetylgalactosamine (UDP-GalNAc) (Wang Z. et al., 2021; Yi et al., 2021; Han et al., 2023; Kadooka et al., 2025). UDP-Glc, UDP-Gal, GDP-Man, and UDP-GlcNAc are crucial fungal cell wall constituents, along with glucan, chitin, galactomannan, and galactosaminoglycans (Yaakoub et al., 2022). Therefore, under dark conditions, the synthesis of *O. apalosarca* cell walls can be promoted, facilitating stipe elongation.

### 4.3 Tyrosinase associated with fungal pigmentation

Caps formed in darkness exhibited white coloration compared with those developed under illumination. This phenomenon has been reported in various mushroom species, including *L. edodes* (Sakamoto, 2018), *Morchella sextelata* (Qiu et al., 2023b), *H. marmoreus* (Jang et al., 2013), and *Auricularia auricula* (Qiu et al., 2023a). Light induces melanin synthesis in mushrooms (Yoo et al., 2019). Most fungi synthesize melanin through the l-DOPA pathway, whose precursor is tyrosine and produces eumelanin (Liu et al., 2022). In the KEGG annotation analysis of *O. apalosarca*, the DEGs in the OaFCS_D and OaFCS_L groups were significantly enriched in the tyrosine metabolism pathway. The tyrosine biosynthesis pathway is associated with fungal browning, as reported in studies on *Agaricus bisporus* (Cai et al., 2022), *F. velutipes* (Fu et al., 2023), *L. edodes* (Flurkey et al., 1994), *H. marmoreus* (Wang G. et al., 2021), and *A. auricula* (Li et al., 2021). Tyrosinase catalyzes the formation of tyrosine and l-DOPA, key metabolic products of the tyrosine-dopamine synthesis pathway, from 4-hydroxy phenylpyruvate aminotransferase. Similarly, through the catalytic action of tyrosinase, tyrosine and l-DOPA are decarboxylated to form dopaquinones. Through oxidation and reaction with tyrosinase, dopaquinone enables the form58 ation of eumelanin (Du et al., 2023). In a comparative transcriptomic study of different cap colors in *P. cornucopiae*, the tyrosinase gene (*PcTYR*) was identified as the candidate gene with the highest confidence for cap color (Zhang et al., 2022). *PcTYR* overexpression resulted in a significantly darker cap color, whereas RNAi exhibited a notably lighter cap color than the wild-type strains, indicating that *PcTYR* plays a crucial role in cap color formation in *P. cornucopiae* (Zhang et al., 2022). Multiple TYR genes have been annotated in *O. apalosarca*, with similar findings in both *L. edodes* (Du et al., 2023) and *P. cornucopiae* (Zhang et al., 2022). Some fungi, such as *P. cornucopiae* and *P. eryngii*, have TYR genes that contain tyrosinase domains, whereas fungi, such as shiitake mushrooms, contain tyrosinase and tyrosinase C domains (Fu et al., 2023). In *O. apalosarca*, a TYR-related gene exists that contains a tyrosinase superfamily domain, and a tyrosinase superfamily domain has been noted in the TYR gene of *Cordyceps militaris*. These findings indicate that these TYR genes in *O. apalosarca* have different origins. The expression level of this gene in *O. apalosarca* was relatively low, indicating that it may have little association with cap-color formation. Genes A6_A10477 and A6_A09603 exhibited relatively high expression levels and were significantly upregulated after light treatment. Therefore, these two TYR-encoding genes may be involved in the regulation of cap color in *O. apalosarca*.

Studies on *O. apalosarca* have limitations, such as unclear life history and genetic patterns, and the lack of a mature genetic manipulation system, making gene function validation challenging. Therefore, establishing a solid theoretical foundation for genetics, building a genetic manipulation system, and conducting gene function research to further clarify the effects of genes such as tyrosinase on its growth and development are necessary. Our study provides support from genomics and transcriptomics data, identifying genes involved in light-induced regulation of its growth and development, thereby laying the foundation for subsequent genetic mechanism research and breeding improvement of *O. apalosarca*.

## 5 Conclusion

*O. apalosarca* possesses significant potential for industrial cultivation, supported by independent intellectual property rights. However, the absence of genomic data has hindered efforts in varietal improvement. Herein, we present the construction of the first genome of *O. apalosarca*, encompassing 53.13 Mb and 27 scaffolds, with N50 and BUSCO values surpassing that of other *Oudemansiella* genomes. Whole-genome phylogenetic and comparative genomic analyses revealed that *O. apalosarca* within the broad genus *Oudemansiella* is closely related to *O. raphanipes* and *M. mucidula*. Under complete darkness, we cultivated pure white, long-stiped fruiting bodies of *O. apalosarca*, advantageous for industrial production. Comparative transcriptome analysis elucidated transcriptional variations during cap and stipe phenotypic changes under both light and dark conditions. DEGs in the caps exhibited a more active cell cycle in light-grown specimens, correlating with larger caps. Concurrently, DEGs related to cell wall formation and carbon metabolism were significantly upregulated in stipes grown in darkness, providing the essential materials and energy for stipe elongation. Tyrosinase-related genes were markedly upregulated in brown caps, implicating their role in melanin synthesis in *O. apalosarca*. This study provides novel insights into the light-induced developmental regulation of *O. apalosarca* and delivers critical genomic and transcriptomic resources to inform future breeding and industrial cultivation methodologies.

## Data Availability

The original contributions presented in the study are publicly available. This data can be found here: Genome Warehouse, National Genomics Data Center, https://ngdc.cncb.ac.cn/gwh, BioProject PRJCA038914.
