# Supplementary material for: First genome report of Oudemansiella apalosarca and comparative transcriptomics on fruiting body formation under different light conditions
Source: Front Fungal Biol. 2026 Feb 10;7:1763719. doi: 10.3389/ffunb.2026.1763719 (PMC12929423; doi:10.3389/ffunb.2026.1763719)
Supplement: Supplementary file 1 [file DataSheet1.docx]

Supplementary Material


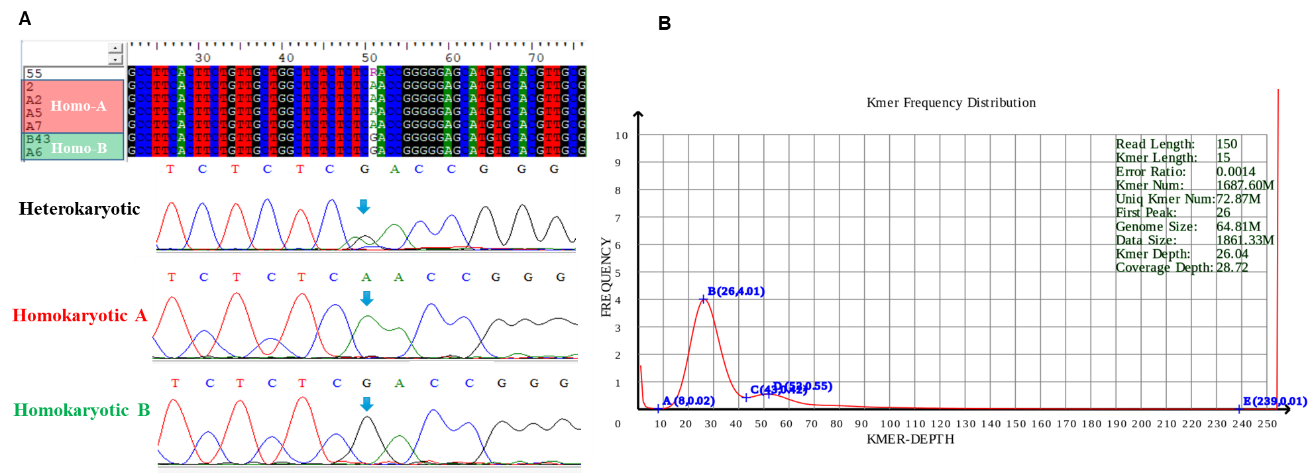


**Figure S1. ITS and survey verification of the native protoplast recovery strain is a homokaryon.** A: ITS analysis. The location indicated by the arrow is the ITS heterozygous site. B: Survey analysis. The survey results indicate that the kimer length is 15, and the heterozygosity is 0.42%.


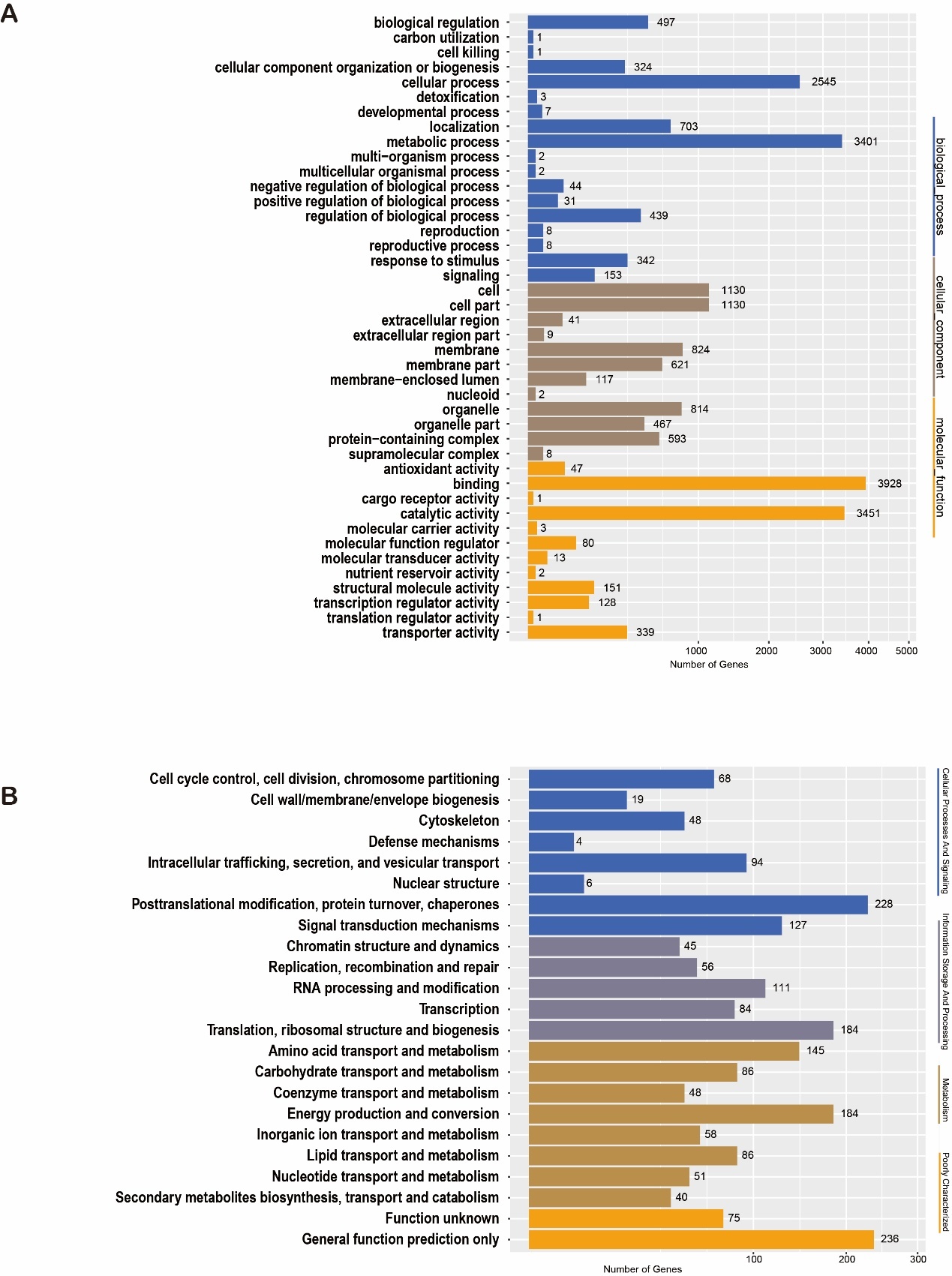


**Figure S2. GO and KEGG annotation of** ***Oudemansiella apalosarca*.** A: GO enrichment analysis of protein cording genes. B: KEGG enrichment analysis of protein cording genes.


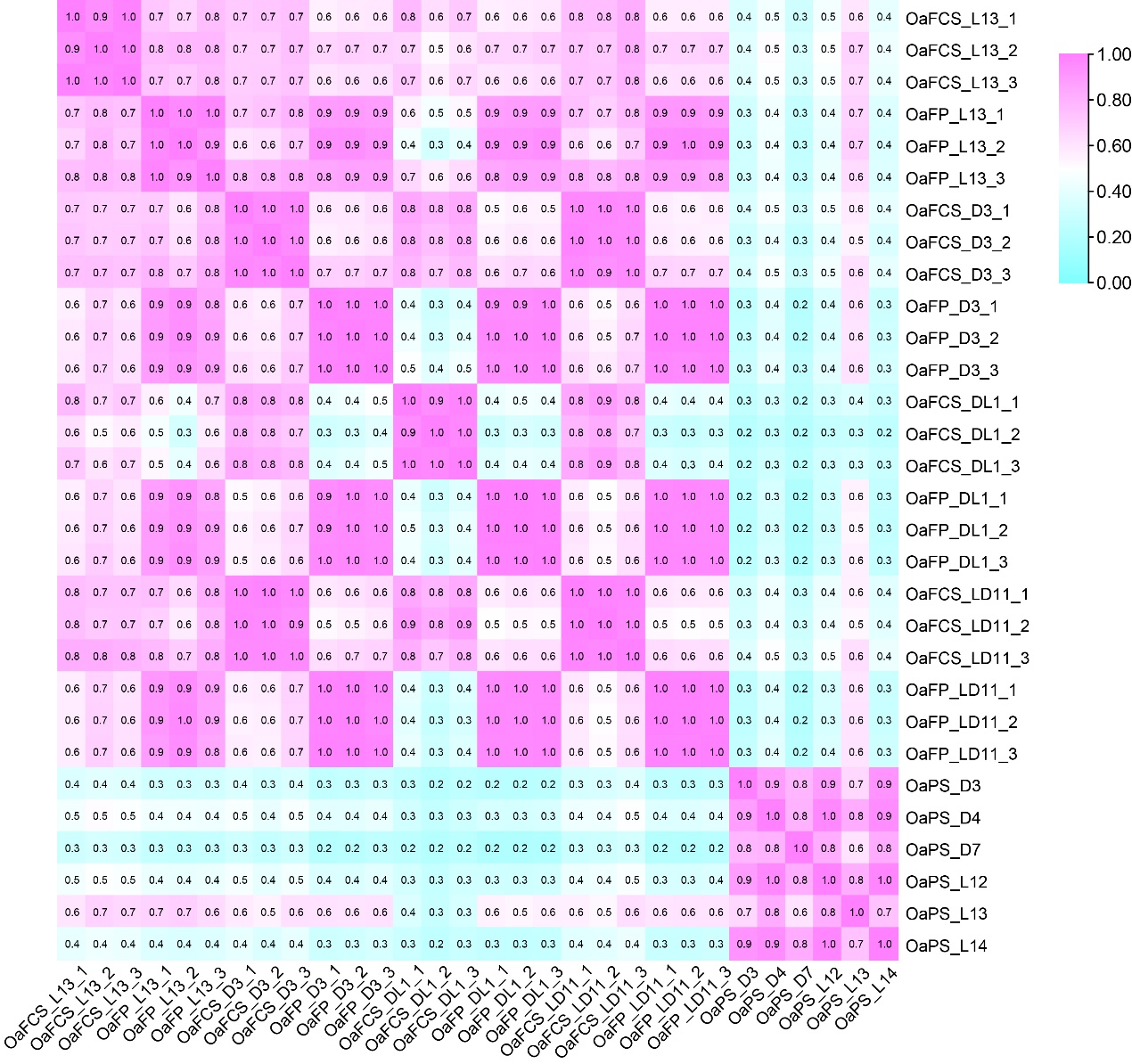


**Figure S3. Pearson correlation value between biological replicates.** The more the color leans towards red, the higher the correlation.


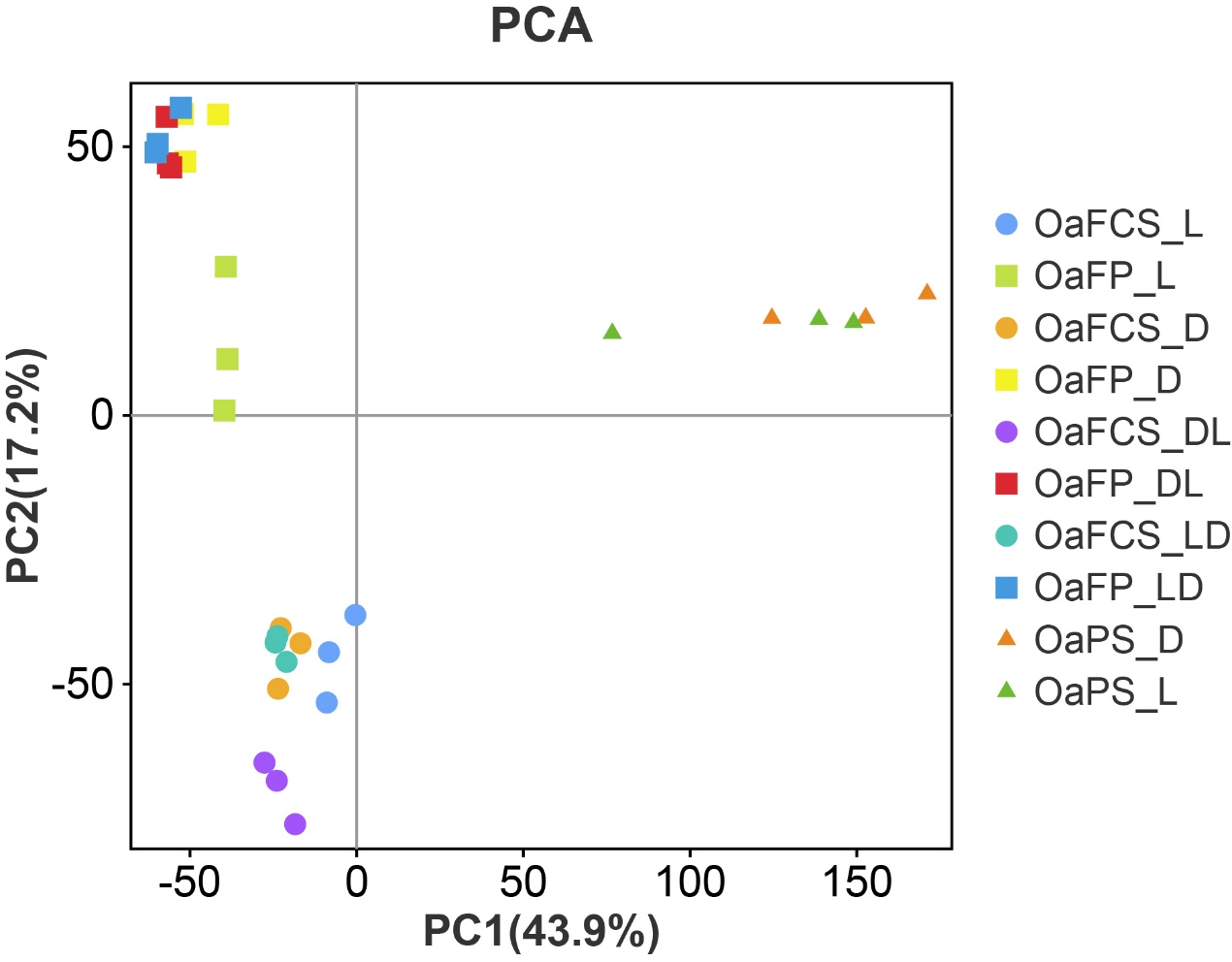


**Figure S4. Principal component analysis (PCA) of all the samples.**


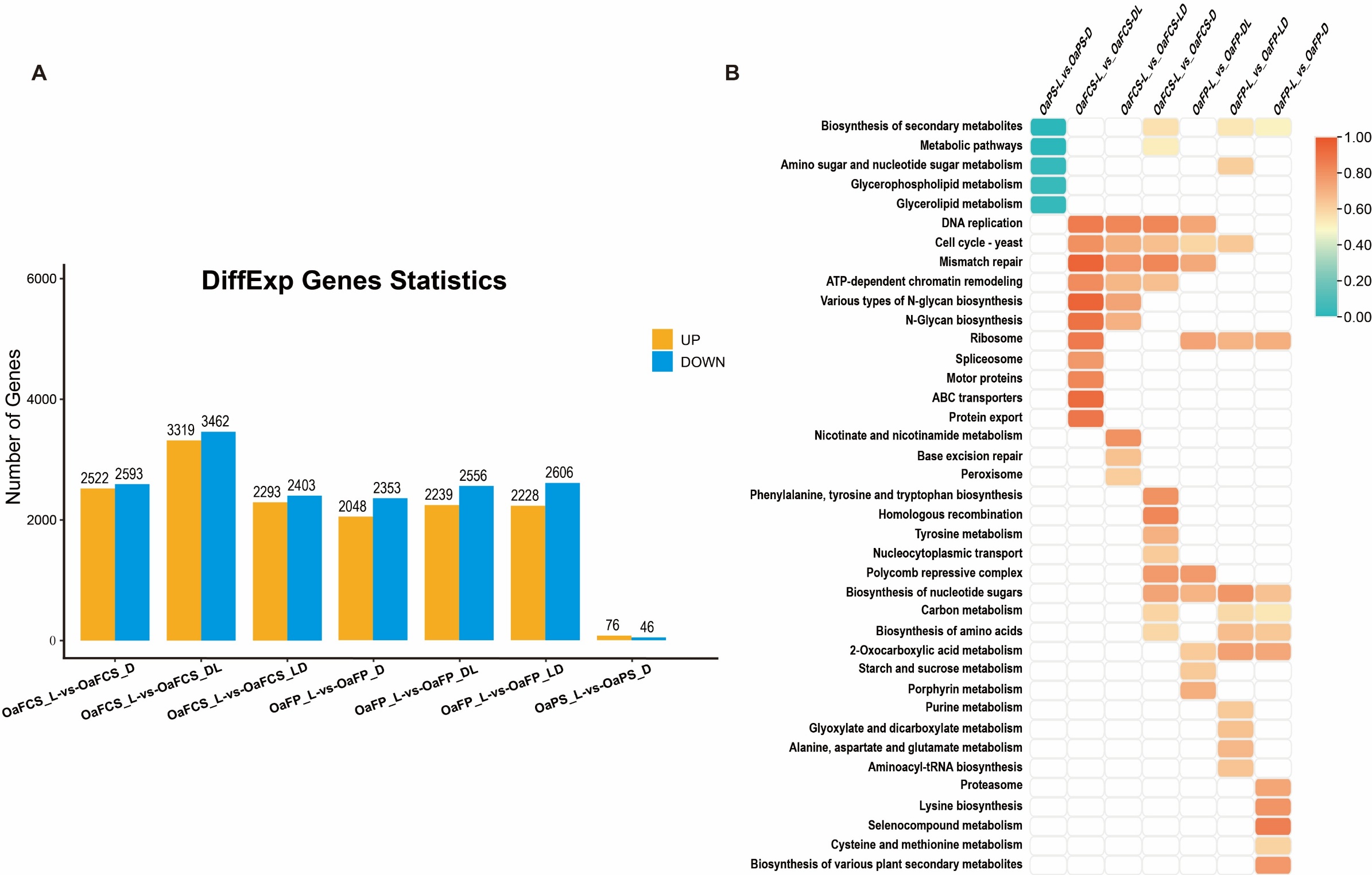


**Figure S5. DEGs among each light treatment.** Orange-yellow represents upregulated expression genes compared to light treatment, while blue represents downregulated expression genes.


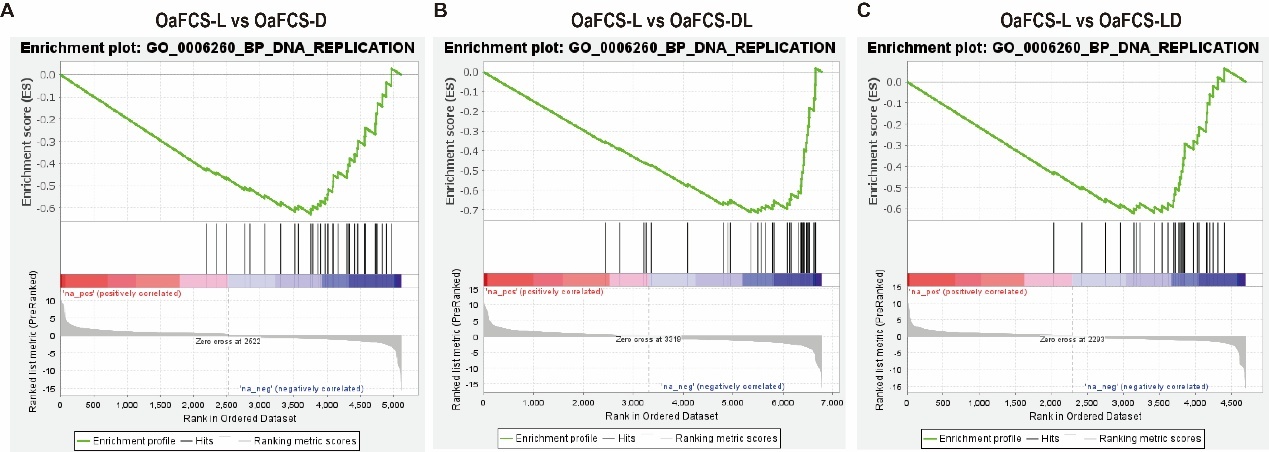


**Figure S6. GSEA** **DNA replication term of DEGS.** A: GSEA DNA replication in OaFCS-L vs OaFCS-D. B: GSEA DNA replication in OaFCS-L vs OaFCS-DL. C: GSEA DNA replication in OaFCS-L vs OaFCS-LD. The DEGs in all three treatments primarily exhibit a trend of downregulation.


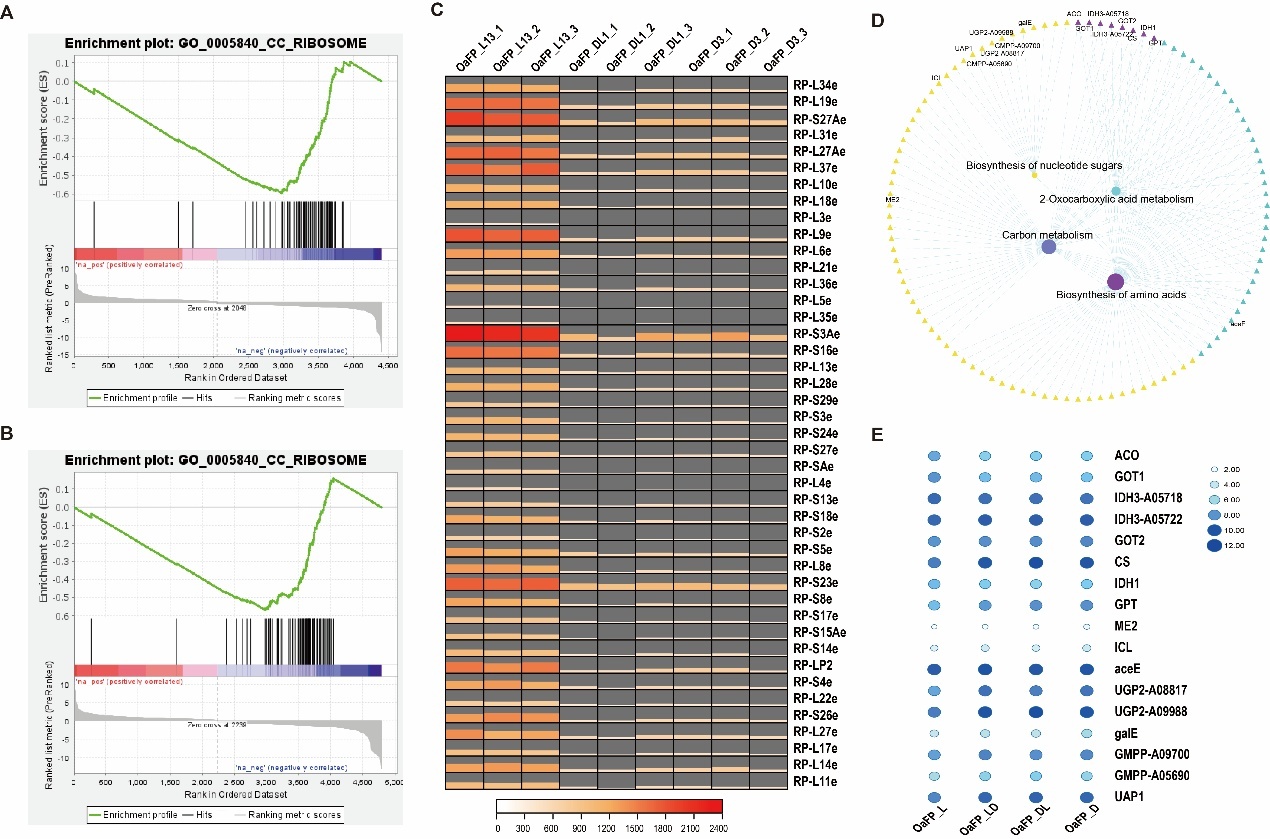


**Figure S7. Expression patterns of common DEGs related to the ribosome pathway and KEGG note DEGs in the stipe.** A: GSEA ribosome term of DEGS in OaFP-L vs OaFP-D. B: GSEA ribosome term of DEGS in OaFP-L vs OaFP-LD. C: Expression patterns of common KEGG and GSEA ribosome DESs in stipe. The x-axis represents the various treatment groups, and the y-axis indicates the expression levels of common DEGs under the corresponding treatment. The redder the color and the taller the bars, the higher the expression levels. D: Net image of commonly enriched KEGG pathways in stipe. The central dots indicate enriched pathways, with larger dots representing a greater number of genes enriched in that pathway. The outer circle displays the related genes that have been enriched; yellow indicates that the gene is enriched in only one pathway, cyan represents that the gene is enriched in two pathways, and mauve signifies that the gene is enriched in three pathways. E: Expression patterns of KEGG note DEGs. The larger and darker the dot, the greater the gene expression level.

**
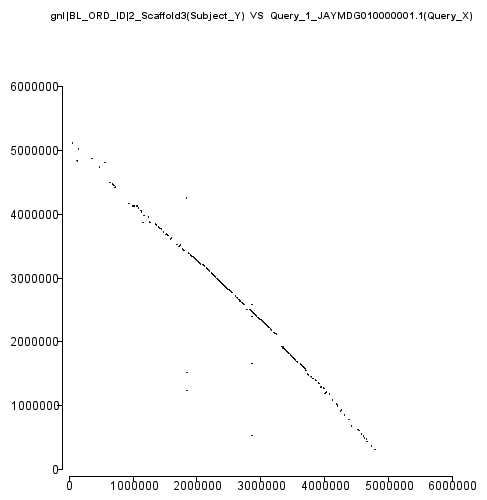
**

**Figure S8. Dot plot of collinearity between A6 strain of *O. apalosarca* and CGG-A-s1 strain of *O. raphanipes***

| **Table S1. The list of tyrosinase from different fungal species** | | |
| --- | --- | --- |
| **Fungal species** | **Gene Bank** | **Definition** |
| *Pleurotus cornucopiae* | UMB36833.1 | tyrosinase |
| *Aureobasidium melanogenum* | KAH0427195.1 | tyrosinase central domain-containing protein, partial |
| *Flammulina filiformis* | XKR89404.1 | tyrosinase |
| *Stropharia rugosoannulata* | XQJ55017.1 | tyrosinase |
| *Agrocybe pediades* | KAF9562472.1 | tyrosinase |
| *Hypsizygus marmoreus* | AIO05492.1 | tyrosinase |
| *Lyophyllum atratum* | KAF8055434.1 | tyrosinase |
| *Macrocybe gigantea* | ATU82297.1 | tyrosinase |
| *Pleurotus eryngii* | KAF9492458.1 | tyrosinase |
| *Aspergillus oryzae* | BAA07149.1 | tyrosinase |
| *Cordyceps militaris* | XP_006669254.1 | tyrosinase |
| *Agaricus bisporus var. bisporus* | XP_006459626.1 | tyrosinase |
| *Ganoderma leucocontextum* | KAI1794979.1 | photo-regulated tyrosinase |
| *Stereum hirsutum* | WVS81704.1 | tyrosinase |
| *Coprinopsis cinerea* | XP_002911630.1 | tyrosinase |
| *Lentinula endodes* | - | tyrosinase |
| *Laccaria bicolor* | XP_001885378.1 | tyrosinase |
| *Armillaria solidipes* | PBK75955.1 | photo-regulated tyrosinase |
| *Mucidula mucida* | KAF8902674.1 | tyrosinase |

**Table S2. Real-time** **PCR primer sequence**

| **Primer name** | **Primer sequence F/ R (5' to 3')** |
| --- | --- |
| actin-F | TATCCACGAGACCACCTACAAC |
| actin-R | ATCCACCAATCCAGACAGAGTA |
| *ARO8*-F | TCATTCCTCCTTCTTCTGGC |
| *ARO8*-R | AGGTGGCAGTAGCCTTCTTA |
| *TYR*-F | CGTTGGTGACGGAGGAAGTT |
| *TYR*-R | CAGAAGGGAGTGAGCGTAGTGT |
| *adhP*-F | ATACTCAGGCGGTGGATTATTT |
| *adhP*-R | CCTTCAAACCCTCGTAGACATT |
| *GGT1-5*-F | AAATAGGCATAGCCGTTCTC |
| *GGT1-5*-R | ATTCTCCTGGTACTGCGACA |
| *GST*-F | CGCTGCCCAATCGTCCGTAG |
| *GST*-R | CCGTCCGACCACAAACATAG |

| **Table S3. Assembly statistics of genome** | | | | | | | | | | | | | | | |
| --- | --- | --- | --- | --- | --- | --- | --- | --- | --- | --- | --- | --- | --- | --- | --- |
| **Sample Name** | **Seq Type** | **Total Number** | **Total Length (bp)** | **N50 Length (bp)** | **N90 Length (bp)** | **Max Length (bp)** | **Min Length (bp)** | **Gap Number (bp)** | **GC Content (%)** | **Complete BUSCOs (%)** | **Complete and single-copy BUSCOs (%)** | **Complete and duplicated BUSCOs (%)** | **Fragmented BUSCOs (%)** | **Missing BUSCOs (%)** | **Total BUSCO groups searched (%)** |
| *O. apalosarca* A6 | Scaffold | 27 | 53133882 | 4308404 | 1072315 | 6095455 | 80247 | 1 | 49.5 | 98.4 | 95.8 | 2.6 | 0.3 | 1.3 | 100 |
| *O. raphanipes* CGG-A-s1 | Scaffold | 37 | 60215257 | 2552576 | 2552576 |  |  |  | 50.2 | 94.4 | 88.7 | 5.7 | 0.9 | 4.7 | 100 |
| *O. radicata* IJFM A160 | contig | 1967 | 78586690 | 104573 | 104573 |  |  |  |  |  |  |  |  |  |  |
| *O. radicata* MG139 | Scaffold | 30341 | 71434061 | 5920 | 6072 |  |  |  |  |  |  |  |  |  |  |
| *O. raphanipes* MG56 | Scaffold | 11311 | 80374122 | 17137 | 17454 |  |  |  |  |  |  |  |  |  |  |

**Table S4. Annotation Statistics**

| **Sample Name** | **Total annotated genes** | **Total Length(bp)** | **Average Length** | **Length/Genome Length (%)** | **SWISSPROT** | **NR** | **KEGG** | **GO** | **IPR** | **KOG** | **OverAll** |
| --- | --- | --- | --- | --- | --- | --- | --- | --- | --- | --- | --- |
| A6 | 14,650 | 26,944,389 | 1,839 | 51 | 2,300 (15.69%) | 9,258 (63.19%) | 3,950 (26.96%) | 6,872 (46.9%) | 9,548 (65.17%) | 1,894 (12.92%) | 11,090 (75.69%) |

| **Table S5. Non-cording RNA statistic** | | | | |
| --- | --- | --- | --- | --- |
| **Type** | **Copy** | **Avg_Lengh** | **Total_Lengh** | **% in Genome** |
| tRNA | 455 | 82 | 37,148 | 0.0699 |
| rRNA(by denovo prediction) | 22 | 2170.54 | 47,752 | 0.0899 |
| sRNA | 31 | 57 | 1,765 | 0.0033 |
| snRNA | 65 | 85.52 | 5,559 | 0.0105 |
| miRNA | 87 | 56.04 | 4,876 | 0.0092 |

| **Table S6. Repeat statistics** | | | | | | | | |
| --- | --- | --- | --- | --- | --- | --- | --- | --- |
| **Method** | **Type** | **DNA** | **LINE** | **LTR** | **SINE** | **Other** | **Unknown** | **Total** |
| Repbase TEs | Length (bp) | 112,984 | 46628 | 947,285 | 2313 | 0 | 1654 | 1,101,227 |
|  | % in Genome | 0.2126 | 0.0878 | 1.7828 | 0.0044 | 0 | 0.0031 | 2.0726 |
| ProteinMask TEs | Length (bp) | 0 | 0 | 0 | 0 | 0 | 0 |  |
|  | % in Genome | 0 | 0 | 0 | 0 | 0 | 0 | 0 |
| Denovo TEs | Length (bp) | 57938 | 0 | 2895822 | 3867 | 0 | 4261393 | 7176128 |
|  | % in Genome | 0.109 | 0 | 5.45 | 0.0073 | 0 | 8.0201 | 13.5057 |
| Combined TEs | Length (bp) | 169,913 | 46628 | 3,233,951 | 6180 | 0 | 4262916 | 7,640,017 |
|  | % in Genome | 0.3198 | 0.0878 | 6.0864 | 0.0116 | 0 | 8.023 | 14.3788 |

| **Table S7. The list of fungal species for evolutionary analysis** | | |
| --- | --- | --- |
| **Fungal species** | **Strain** | **Gene Bank** |
| *Lentinula edodes* | L808 | GCA_015476405.1 |
| *Gymnopus luxurians* | FD-317 M1 | GCA_000827265.1 |
| *Armillaria solidipes* | 28-4 | GCA_002307675.1 |
| *Cylindrobasidium torrendii* | FP15055 ss-10 | GCA_000934385.1 |
| *Flammulina velutipes* | 6-3 | GCA_011800155.1 |
| *Oudemansiella radicata* | IJFM A160 | GCA_015501595.1 |
| *Mucidula mucida* | CBS 558.79 | GCA_015501055.1 |
| *Coprinopsis cinerea* | okayama7#130 | GCF_000182895.1 |
| *Stropharia rugosoannulata* | A15 | GCA_036873085.1 |
| *Agrocybe pediades* | CBS 102.39 | GCA_013053245.1 |
| *Laccaria bicolor* | S238N-H82 | GCF_000143565.1 |
| *Volvariella volvacea* | WC 439 | GCA_001691835.3 |
| *Agaricus bisporus* | H119_p4 | GCA_014872705.1 |
| *Hypsizygus marmoreus* | 51987-8 | GCA_001605315.2 |
| *Pleurotus ostreatus* | PC9 | GCA_014466165.1 |
| *Pleurotus eryngii* | ATCC 90797 | GCA_015484515.1 |
| *Schizophyllum commune* | H4-8 | GCF_000143185.2 |
| *Ganoderma lucidum* | G.260125-1 | GCA_000271565.1 |
| *Sparassis crispa* | - | GCF_003851025.1 |
| *Stereum hirsutum* | FP-91666 SS1 | GCA_000264905.1 |
| *Hericium alpestre* | DSM 108284 | GCA_004681135.1 |
| *Tremella mesenterica* | Fries | GCA_000271645.1 |
| *Cordyceps militaris* | CM01 | GCA_000225605.1 |
| *Saccharomyces cerevisiae* | S288C | GCA_000146045.2 |

| **Table S8. Different treatments correspond to *Oudemansiella apalosarca* samples** | |
| --- | --- |
| **Treatment** | **Samples** |
| Cap Skin of Fruiting body in light | OaFCS-L13-1 |
|  | OaFCS-L13-2 |
|  | OaFCS-L13-3 |
| Stipe of Fruiting body in light | OaFP-L13-1 |
|  | OaFP-L13-2 |
|  | OaFP-L13-3 |
| Cap Skin of Fruiting body in dark | OaFCS-D3-1 |
|  | OaFCS-D3-2 |
|  | OaFCS-D3-3 |
| Stipe of Fruiting body in dark | OaFP-D3-1 |
|  | OaFP-D3-2 |
|  | OaFP-D3-3 |
| Cap Skin of Fruiting body in dark-light treatment | OaFCS-DL1-1 |
|  | OaFCS-DL1-2 |
|  | OaFCS-DL1-3 |
| Stipe of Fruiting body in dark-light treatment | OaFP-DL1-1 |
|  | OaFP-DL1-2 |
|  | OaFP-DL1-3 |
| Cap Skin of Fruiting body in light-dark treatment | OaFCS-LD11-1 |
|  | OaFCS-LD11-2 |
|  | OaFCS-LD11-3 |
| Stipe of Fruiting body in light-dark treatment | OaFP-LD11-1 |
|  | OaFP-LD11-2 |
|  | OaFP-LD11-3 |
| Small Primordial in dark environment | OaPS-D3 |
|  | OaPS-D4 |
|  | OaPS-D7 |
| Small Primordial in light environment | OaPS-L12 |
|  | OaPS-L13 |
|  | OaPS-L14 |

| **Table S9 Data summary of RNA-seq** | | | | | | | | |
| --- | --- | --- | --- | --- | --- | --- | --- | --- |
| **Sample id** | **Raw reads** | **Raw bases(G)** | **Clean reads** | **Total mapped** | **Unique mapped** | **Q20(%)** | **Q30(%)** | **GC Content(%)** |
| OaFCS_L13_1 | 44,031,232 | 6.6 | 42,288,586 | 85.56% | 78.02% | 94.2 | 85.3 | 52.8 |
| OaFCS_L13_2 | 77,031,802 | 11.55 | 74,267,692 | 86.64% | 79.19% | 94.4 | 85.7 | 53 |
| OaFCS_L13_3 | 61,323,454 | 9.2 | 58,577,976 | 85.95% | 78.89% | 94.9 | 86.6 | 53.1 |
| OaFCS_LD11_1 | 56,829,088 | 8.52 | 54,481,436 | 86.32% | 79.31% | 94.9 | 86.8 | 52.8 |
| OaFCS_LD11_2 | 53,984,310 | 8.1 | 51,617,838 | 85.94% | 79.25% | 95.3 | 87.6 | 52.8 |
| OaFCS_LD11_3 | 45,073,046 | 6.76 | 42,175,240 | 85.15% | 78.53% | 95.4 | 87.9 | 52.7 |
| OaFCS_DL1_1 | 59,987,672 | 9 | 55,488,824 | 83.45% | 76.53% | 95.5 | 88.2 | 52.6 |
| OaFCS_DL1_2 | 61,016,070 | 9.15 | 56,662,122 | 83.95% | 77.10% | 95.8 | 88.9 | 52.4 |
| OaFCS_DL1_3 | 68,656,304 | 10.3 | 63,638,632 | 83.91% | 77.09% | 95.9 | 89 | 52.5 |
| OaFCS_D3_1 | 73,738,112 | 11.06 | 71,073,430 | 86.90% | 80.11% | 95.1 | 87.2 | 52.8 |
| OaFCS_D3_2 | 82,035,264 | 12.31 | 77,450,008 | 85.29% | 78.20% | 94.9 | 86.7 | 52.6 |
| OaFCS_D3_3 | 46,524,466 | 6.98 | 44,476,958 | 86.83% | 80.04% | 95 | 87 | 52.8 |
| OaFP_L13_1 | 62,151,610 | 9.32 | 59,845,354 | 86.36% | 78.80% | 94.8 | 86.5 | 53.2 |
| OaFP_L13_2 | 104,270,808 | 15.64 | 99,894,020 | 87.35% | 80.33% | 95.2 | 87.4 | 53.4 |
| OaFP_L13_3 | 65,669,374 | 9.85 | 62,230,548 | 85.87% | 78.92% | 95.1 | 87.2 | 53.3 |
| OaFP_LD11_1 | 66,933,378 | 10.04 | 61,742,840 | 83.77% | 76.57% | 95.5 | 88.2 | 53.2 |
| OaFP_LD11_2 | 71,953,656 | 10.79 | 67,642,978 | 86.25% | 79.40% | 95.5 | 88.3 | 53.3 |
| OaFP_LD11_3 | 46,374,614 | 6.96 | 44,087,956 | 85.40% | 78.03% | 94.6 | 86.1 | 53.1 |
| OaFP_DL1_1 | 68,301,012 | 10.25 | 65,493,696 | 86.39% | 79.01% | 94.8 | 86.4 | 53.3 |
| OaFP_DL1_2 | 43,262,392 | 6.49 | 41,086,792 | 85.35% | 77.90% | 94.7 | 86.3 | 53.2 |
| OaFP_DL1_3 | 52,555,268 | 7.88 | 49,992,362 | 85.96% | 78.65% | 94.8 | 86.4 | 53.2 |
| OaFP_D3_1 | 73,951,492 | 11.09 | 67,419,876 | 83.08% | 76.19% | 96 | 89.3 | 53.2 |
| OaFP_D3_2 | 42,435,220 | 6.37 | 39,716,712 | 84.14% | 76.63% | 94.2 | 85.2 | 53.1 |
| OaFP_D3_3 | 55,440,552 | 8.32 | 52,757,610 | 86.04% | 79.21% | 95.1 | 87.1 | 53.2 |
| OaPS_L12 | 68,695,420 | 10.3 | 65,738,744 | 85.03% | 78.33% | 95.2 | 87.6 | 53 |
| OaPS_L13 | 75,902,222 | 11.39 | 71,835,444 | 85.37% | 78.73% | 95.6 | 88.3 | 53.2 |
| OaPS_L14 | 44,412,814 | 6.66 | 42,340,500 | 85.37% | 78.09% | 95.2 | 87.5 | 52.9 |
| OaPS_D3 | 92,276,976 | 13.84 | 88,212,136 | 85.08% | 78.00% | 95.1 | 87.2 | 53.1 |
| OaPS_D4 | 69,632,156 | 10.44 | 65,780,782 | 85.25% | 78.60% | 95.5 | 88.2 | 53.1 |
| OaPS_D7 | 58,974,128 | 8.85 | 56,421,900 | 85.79% | 78.92% | 95 | 86.9 | 52.8 |
